# Comparing the Rate-All-That-Apply and Rate-All-Statements Question Formats across Five Countries

**DOI:** 10.3390/foods10040702

**Published:** 2021-03-25

**Authors:** Denis Richard Seninde, Edgar Chambers

**Affiliations:** Center for Sensory Analysis and Consumer Behavior, Kansas State University, Manhattan, KS 66502, USA; seninde@ksu.edu

**Keywords:** check-all-that-apply (CATA)‎, rate all that apply (RATA)‎, rating, rate all statements, check all statements, survey, sensory, marketing

## Abstract

Rate All That Apply (RATA) is a derivative of the popularly used Check-All-That-Apply (CATA) question format. For RATA, consumers select all terms or statements that apply from a given list and then continue to rate those selected based on how much they apply. With Rate All Statements (RATING), a widely used standard format for testing, consumers are asked to rate all terms or statements according to how much they apply. Little is known of how the RATA and RATING question formats compare in terms of aspects such as attribute discrimination and sample differentiation. An online survey using either a RATA or RATING question format was conducted in five countries (Brazil, China, India, Spain, and the USA). Each respondent was randomly assigned one of the two question formats (*n* = 200 per country per format). Motivations for eating items that belong to five food groups (starch-rich, protein-rich, dairy, fruits and vegetables, and desserts) were assessed. More “apply” responses were found for all eating motivation constructs within RATING data than RATA data. Additionally, the standard indices showed that RATING discriminated more among motivations than RATA. Further, the RATING question format showed better discrimination ability among samples for all motivation constructs than RATA within all five countries. Generally, mean scores for motivations were higher when RATA was used, suggesting that consumers who might choose low numbers in the RATING method decide not to check the term in RATA. More investigation into the validity of RATA and RATING data is needed before use of either question format over the other can be recommended.

## 1. Introduction

In quantitative consumer research, several question formats are used to collect respondents’ product descriptions and perceptions, opinions, beliefs and attitudes (POBAs). Questionnaires for consumer studies (e.g., online surveys, central location tests, and home-use tests) may include the highly popular Check-All-That-Apply (CATA) [1,2,3], or Check-All-Statements (CAS) [2,4,5,6], or RATING questions [7,8,9,10], or the Rate-All-That-Apply (RATA) questions [3,11,12,13,14].

For the Check-All-That-Apply (CATA) or Mark-All-That-Apply format, respondents select all attributes or statements that apply from a given list. Easy and non-tedious are the key reasons why CATA has gained popularity in recent years [2,15,16,17,18]. However, there is criticism of this question format because of the equivocal interpretations of the unchecked attributes on the listed options [1,4,5,19]. Conversely, the Check-All-Statements (CAS) or the forced-choice yes/no questions require respondents to provide a response (e.g., yes/no or agree/disagree) for each attribute or statement to show that it applies or does not apply. Although CAS is immune to primacy bias (attributes at the top of the list are marked more frequently than those at the bottom of the list), which is prevalent with CATA questions, CAS has been associated with acquiescence bias, where respondents tend to mark or agree with the positive connotation for all survey questions [1,4,20,21,22].

### 1.1. Rate All Statements (RATING)

The Rate-All-Statements (RAS) or simply the RATING question format uses intensity, or degree, scales to rate consumers’ responses for each attribute or term in relation to the particular product(s) that are being investigated [7,9]. Cognitive psychologists and other consumer researchers have for over five decades considered the RATING question format as the gold standard for measuring the intensity or degree of importance or applicability of product attributes [8,23,24,25,26,27]. Unipolar and bipolar scales (i.e., present different degrees of one attribute and another set of degrees of the opposite attribute) are usually used with the RATING question format [28,29,30,31,32]. Lengths of these intensity scales can vary depending on the objective of the consumer study and the desired level of scale sensitivity or discrimination [28]. For example, an intensity scale can have 5 points, i.e., not at all important, slightly important, moderately important, very important, and extremely important, but can also be shorter, with just 3 points (low, medium, and high) [3,12].

Stevens [26] recommended that interval-scale data such as intensity, degree, or Likert scales can be analyzed using parametric tests which comprise arithmetic means with t-tests or analysis of variance (ANOVA) to determine significance. The numerical value assigned to each node of the intensity scale allows for linear transformation of the data without loss of information [26]. However, treating these ordinal scales as interval scales has been met with controversy by various authors [33,34,35,36,37] who advocate for the use of non-parametric tests (e.g., chi-square tests and Spearman’s Rho) to analyze the ordinal data. One advantage of the RATING question format is that its scales collect more detail and also provide for ranking of respondents’ opinions, something that is not the case for other question formats such as CAS and CATA question formats, where respondents provide either a yes/no response and a simple check for those terms that apply, respectively. Further, scales used for RATING questions are usually anchored on one end, with terms such as “strongly disagree”, “never”, “none” and “not important at all” which give the respondent an “out” in case they do not find the particular term or attribute important or applicable to the product that is being examined. One notable disadvantage for the RATING question format is that accuracy of responses could be impacted based on the subject or topic being assessed. For instance, it is possible that respondents may provide incorrect responses to socially sensitive questions (e.g., child abuse behavior and sexual habits) [38]. Further, considering that the RATING question format requires a greater thought process than other question formats such as CATA and CAS, it is possible that the consumers’ survey mean duration could be longer, which could impact the cost of consumer studies, since more time means more money. Similarly, survey incompletion rates (non-response error) for the RATING format could be higher than those of other question formats such as CATA and CAS [39]. Nevertheless, the RATING question format remains the unofficial gold standard for product description questioning in consumer studies [23].

### 1.2. Rate All That Apply (RATA)

For the Rate-All-That-Apply (RATA) question format, after checking all terms that “apply” from a list of options (that is a CATA question), respondents are asked to rate them using a scale that can be 3–9 points [12,13,14,40,41]. Put simply, RATA is a combination of the CATA and RATING question formats [14,41]. Ng et al. [41] noted that although the CATA question format has become highly popular in recent years because of its ease and non-tedious structure, its degree of discrimination among samples, particularly among samples of similar profiles, is limited. This inspired the development of a spin-off question format which saw the inclusion of an intensity or degree scale (e.g., 3-point or 5-point scale) onto the CATA question structure [14]. Data collected using RATA questions can be analyzed in two ways. The first method involves treating of RATA responses as CATA data and conducting analyses such as correspondence analysis. The second and recommended way of analyzing RATA data is by treating it as interval-scale data rather than ordinal data [3,12]. As such, unchecked attributes could be coded with a zero. Meyners et al. [12] explains that if a 3-point scale was used to rate the checked terms, it could be treated as a 4-point scale and analyzed using parametric tests such as t-test and analysis of variance (ANOVA). Ideally, RATA would be expected to benefit from the best features of CATA and RATING, i.e., fairly easier to complete with a lesser burden to respondents than RATING, and enhanced sample discrimination and a more detailed sample description capability than CATA. Additionally, the RATA question format could be associated with CATA limitations such as primacy bias and ambiguity in interpreting unchecked attributes when RATA is used in place of RATING. Vidal et al. [3] who compared CATA and RATA data collected from seven consumer studies found that RATA data were not superior than CATA data. In fact, those authors stated that the use of RATA instead of CATA could be influenced by the overall research objective and the particular sample or product characteristics [3,14]. At the time of writing, there is no research that compared RATA and RATING data in terms of aspects such as discrimination among samples and discrimination among attributes, non-response error (survey incompletion rates) which are important parameters that researchers use determining what question format to use in future consumer studies.

Rapid advancements in information technology in recent years—for example, the increased access to faster and affordable internet in East Asia, North America and Western Europe—have made online or web surveys a popular survey method for consumer researchers in multiple countries [42,43,44,45]. Online surveys are a cheaper, faster, and far-reaching (larger numbers of respondents) data collection option than other survey methods such as in-person interviews, telephone interviews or mail surveys. Additionally, the fact that several features can be added to online survey designs (these can include videos, audio clips, and product nutrition information labels) has made online surveys a staple for many consumer researchers [46]. Conrad et al. [47] suggested that inclusion of dialog-like features in web survey designs could improve respondents’ understanding of survey questions and accuracy of collected responses. The RATA question format is one possible way of including human dialogue to online surveys.

The overall objective of the current writing was thus to examine the characteristics of data that were collected using the RATA and RATING question formats in an online survey. Comparison of RATA data with data collected using the gold-standard method could provide better understanding of when researchers could use the RATA question format. Additionally, five versions (five languages/countries) of this online survey were conducted to assess the consistency or replicability of the data characteristics for the two question methods. Specific objectives for the questionnaire comparisons were to (a) compare the percentage of “apply” responses for RATA and RATING; (b) compare response distribution based on ratios of “apply” responses; (c) compare the mean scores for eating motivation constructs or terms for each food category within five countries; (d) identify the level of importance accorded to constructs by RATA and RATING question formats; (e) compare the significant differences among food categories or samples for RATA and RATING; (f) compare consumers’ survey mean duration, survey liking, just-about-right (JAR) rating, and completion rates for RATA and RATING survey formats.

## 2. Materials and Methods

### 2.1. Survey Structure

An eating motivation survey (EMS) which included questions on consumers’ motivations for eating or not eating food items that belong to five food groups was used for this online study [1,7]. The questionnaires were randomly assigned to respondents in either the RATA or RATING formats but not both (each respondent saw only one format). A total of 47 positive motivation terms that could be categorized into 16 eating motivation constructs were assessed in each question format of the EMS [1,7]. Each eating motivation construct consisted of three terms or subscales except for the choice limitation construct that had only two terms. Details on why the authors used the 47 motivation terms and how “apply” responses for the subscales were summarized into 16 constructs has been published previously [1,7]. For the RATA question format, the 47 terms were randomized for each respondent. Additionally, respondents marked the terms that applied and continued to rate how much each of the checked terms applied based on a five-point intensity type scale. The scale was anchored with “not at all important” at one end and “extremely important” at the other with internodes of “slightly important”, “moderately important”, and “very important”. The RATING question format, on the other hand, did not provide an option for the respondents to check what terms applied but rather asked them to rate the level of importance or applicability of each of the 47 terms based on the same five-point Likert intensity scale that was used for the RATA format. RATING questions were not randomized for each respondent. For RATA, all 47 items were presented on a single page for the respondent to Check All That Apply followed by separate pages for individually rating each of the selected terms. As for the RATING question format, five terms were assessed on a single page because of computer screen page limitations. These formats are typical of many on-line or computer-based consumer studies using RATA or rating. The number of respondents and number of terms or attributes that were assessed in the current survey was not unusual. In fact, the literature shows several articles where a similar number of terms or attributes were evaluated [2,4,48,49,50].

The subject for survey questionnaires was consumers’ motivations for eating items that belonged to five food groups. The food groups included foods rich in starch (e.g., potato and rice dishes), proteins (e.g., meat, beans), dairy, fruits, and sweet foods/desserts [48,51]. Authors used food items that fit in these food groups and were relevant to the particular country [7]. For example, in all countries, bananas were used as the fruit. In the case of starch-rich foods, baked potatoes were used for the USA, while paella was used for Spain and white rice was used for Brazil, China, and India. These foods were chosen based on discussions with multiple sensory scientists in each country who reviewed and discussed all the foods chosen in all countries to ensure the products represented the “concept” of the food category as much as possible for that country. Where possible, similar foods were used (e.g., white rice in three countries for “starch-rich foods”), though where the product was not widely consumed in that form (e.g., Spain) or not consumed in a similar form by a large percentage of the population (e.g., USA), alternative products were selected that were more commonly eaten.

The online survey questionnaire also included other questions that were included in the survey timing. For example, two questions that investigated the respondents’ survey experience in terms of respondent liking (a hedonic question) and a rating question based on the perceived length of the survey (a just-about-right or JAR question) were included near the end of the survey. The respondents’ survey liking question and the JAR question were each placed on separate pages. The two survey questionnaires were initially written in English for the respondents in the USA. The approved survey question formats written in American English were then translated into Simplified Mandarin, Hindi, Spanish, and Portuguese for respondents in China, India (English also provided as an option), Spain, and Brazil, respectively. The survey translation process used a variation of the translation, review, adjudication, retesting, and documentation (TRAPD) approach [52,53]. The full procedure for the survey methods, including translation, and the surveys in all five languages have been published previously [7].

All subjects gave their informed consent for inclusion before they participated in this online survey. Additionally, the survey was conducted in accordance with the Declaration of Helsinki, and the protocol was approved by the designated review board at Kansas State University, Manhattan, KS, USA subjects (IRB #7297.2).

### 2.2. Respondents and Recruitment

Respondents in five countries were recruited by Qualtrics, Provo, UT, USA using its or its partners’ existing databases. Using the Qualtrics survey software, one format of the survey questionnaire with RATA questions and another with RATING questions were assigned randomly to 400+ respondents per country (*N*~200 per questionnaire per country) [7]. Respondents were required to be 18 or older and then were recruited to fill demographic quotas of age and gender for each questionnaire format (RATA and RATING). Four age groups (*n* = 50+ per age group) were used in this study: Generation Z (born in the years 1995 to 2001), millennials (born in the years 1980 to 1994), Generation X (born in the years 1965 to 1979) and baby boomers or boomers (born in the years 1944 to 1964). For each age group, 50% were female and 50% were male. Once the required number of completed responses for a particular quota was filled, newly qualified respondents (for the filled quotas) were discontinued from completing the EMS. Other demographic data that were collected for informational purposes included respondents’ level of education and number of adults and number of children in the households (Table 1).

The respondents in both samples were selected with “matched” demographics of age and gender within each country. The sample sizes are also reasonably large for each group (>200 per group in each of the five countries) and no characteristic (gender, age, education, household numbers) was significantly different between the two samples from each country. Thus, we conclude that any differences noted between the two question formats are likely driven by the formats and not some inherent bias among the respondents.

### 2.3. Data Analysis

#### 2.3.1. Comparison of Percentages of “Apply” Responses

Consumers’ responses were categorized into two baskets. The first basket was the “apply” basket that included RATA and RATING responses, where consumers rated the motivation terms or subscales as important such as either “slightly important” or “moderately important” or “very important” or “extremely important” to them eating the particular items that belonged to the different food groups. The second basket included the “not at all important” or “not apply” responses for the RATING survey questions. Additionally, this basket consisted of responses for cases where respondents on second thought rated a term as “not at all important” even though they had previously checked it as “apply”. Data analyses such as comparisons between RATA and RATING question formats in the current writing focused on the “apply” responses, e.g., percentages of “apply” responses, standard indices for RATA and RATING, and ratios of RATING to RATA.

The percentages of “apply” responses for RATA and RATING for all 16 motivation constructs for all five food groups for all countries were calculated. Additionally, although the current paper focused on comparisons between RATA and RATING data, Check-All-That-Apply (CATA) “apply” percentages based on RATA data were also calculated. Percentages were used because the possible number of ticks/checks varied depending on how many people ate that particular food in a particular country and the number of subscales in the eating motivation category.

#### 2.3.2. Establishment of Standard Indices for RATA and RATING and Ratios of RATING to RATA

Standard indices of importance (SII) of “apply” responses were determined for all motivation constructs versus liking within RATA or RATING survey formats. The standard index of importance is an index value that shows the proportion of the number of “apply” responses for any motivation construct to “apply” responses for the liking motivation [1]. Earlier studies [1,48,49] have shown the Liking construct to be the highest motivation on average. Using liking as the comparison index factor (the denominator in the proportion calculation) within each food group, country, and consumer demographic segment allows a within-sample “variable” to be used to adjust all comparisons and put them on a similar “scale” (typically 0–1.0) [1]. Note, it is possible to exceed 1.0 when a motivation exceeds liking in importance for a group of consumers, although this rarely happened. Put simply, the SII is 1.0 when the “apply” responses for any motivation are equal to the “apply” responses for liking within that method for that group of respondents. Similarly, the SII would be 0.5 when a motivation response is half the number compared to liking and so forth. The index was created using the principles espoused by creators of other indices for psychological phenomena that must be compared across various segments though can vary in response behavior across segments [54,55]. If the RATA and RATING formats were assessing the same behavioral patterns of consumers, then the SII values for CAS and CATA for the different motivation constructs would be similar or relatively close. However, if the SII values for the two formats were different, this would indicate that the questions from the two formats were interpreted, processed, and answered differently by the respondents. Major differences in standard index values for motivations within RATA or RATING would suggest that the results of the two survey formats likely would provide different information to the consumer researchers. Such findings would suggest that RATA and RATING, for various reasons, do not measure the same psychological phenomena or, at a minimum, the results would be interpreted differently [1].

Further, the ratios of percentages of “apply” responses for RATA to RATING were calculated to determine whether the ratio of responses varied or remained the same between the two survey formats.

#### 2.3.3. Comparison of Mean Scores for All Eating Motivation Constructs

Meyners et al. [12] recommended the use of mean scores in the analysis of RATA data as opposed to analyzing RATA data as CATA. Two issues had to be addressed when using the RATA rating data. First, in cases where respondents changed their mind, i.e., the respondent checked a motivation term as “apply” but then rated it as “not at all” (suggesting that they should not have checked the term to begin with), that specific data point was included in the analysis as a “1”. Second, in cases where none of the motivations within a construct (usually three motivation subscales per construct) were checked by a respondent, a score of “1” (not at all important) was used in the analysis of the overall construct for that consumer.

Two-sample *t*-tests were used to compare mean scores for RATA and RATING responses for all 16 constructs for all food categories in all five countries with a significance level of *p* ≤ 0.05 [3]. Significant differences (*p* ≤ 0.05) between means of a particular motivation construct would indicate that not only did the consumers interpret, process and answer the respective questions differently but also the interpretation by researchers could be different.

#### 2.3.4. Identification of the Level of Importance for Motivation Constructs

Motivation constructs whose percentage of “apply” responses made the list of top five for the RATA or RATING survey formats for each country for all the five food categories were identified [1]. Similarly, motivation constructs whose mean scores for RATA and RATING were in the top five positions within each food category and each country were also identified.

#### 2.3.5. Comparing Significant Differences among Food Categories

Analysis of variance (ANOVA) at a 5% level of significance was used to identify significant differences among the food categories [3,12]. Post hoc mean separation was carried out using Fisher’s Least Significant Difference (LSD). This was performed to determine which of the two question formats showed better discrimination ability among samples.

#### 2.3.6. Comparison of Survey Format Completion Rates, Survey Mean Duration, Survey Liking, and Survey JAR Rating

Percentages of completion rates for consumers who answered either RATA or RATING question formats of the survey were calculated. Additionally, chi-square tests at a 5% level of significance based on counts of incomplete responses for each format in each country were computed. Additionally, two-sample *t*-tests at a 5% level of significance were computed to provide comparisons of survey format means and standard deviations for consumers’ survey mean duration, survey liking, and survey JAR rating for each country.

All analyses were run using XLSTAT (version 2020.1, AddinSoft, New York, NY, USA).

## 3. Results and Discussion

### 3.1. Comparison of Percentages of “Apply” Responses

In this paper, the term “apply” refers to (a) responses for which the respondents selected motivation terms (in RATA) or (b) marked responses for “extremely important”, “very important”, “moderately important” and “slightly important” for either RATA or RATING survey formats. The RATING question format was associated with a significantly higher percentage of “apply” responses for all 16 motivation constructs for all five food groups in all countries as compared to corresponding CATA and RATA data. For example, In Brazil, CATA and RATA question formats showed that 48% and 47% of respondents, respectively, identified habits as an important motivation for eating starch-rich foods while RATING showed that 94% of corresponding respondents identified the habits construct as important (Table 2). Data for protein-rich foods, dairy foods, fruits and vegetables and dessert foods are presented in Appendix A Table A1, Table A2, Table A3 and Table A4. A similar case was seen in China, where 80% of RATING question format respondents identified visual appeal as an important motivation for eating white rice (a starch-rich food), whereas only 4% and 3% of CATA and RATA responses, respectively, identified the same construct as important. Seeing that “visual appeal” of starch-rich foods garnered a higher frequency in RATING than RATA in China implies that it may be more important than the RATA or CATA suggest. It is also possible that either the RATING questions overestimated the level of importance of the visual appeal construct or that the RATA questions underestimated the level of importance of the same construct to the respondents.

The higher percentage of “apply” responses for the RATING survey format was expected based on multiple aspects, but has important implications for researchers. When consumers show that a term or construct is more applicable in one method than another, that shows that the method impacts the interpretation of the information. For example, in the data shown in Table 2, only approximately one-quarter of consumers (or less in some countries) using the RATA format indicated that eating starch-rich foods (i.e., rice or potatoes) was motivated by health concerns. In contrast, for the RATING format, more than three-quarters of consumers in each country indicated that eating such foods was motivated by concerns related to “health”. Those findings bring vastly different conclusions about the importance of “health” in selecting such foods. Product developers, sensory and marketing scientists, and nutrition and health professionals would use different strategies to encourage or discourage such consumption depending on which method was used for the research. That points to a major problem and discrepancy that needs to be addressed before a decision is made regarding survey methods. Which method is correct? We cannot know from this research and further investigation is needed.

This suggests that the differences may be an artifact of the testing methodology, either from difference in the psychological “threshold” of importance used by consumers in the various methods or in various biases that may be inherent in the methods. RATA respondents checked only terms that “applied” or were important and then continued to rate the level of importance of the selected terms. Not checking a term could be the result of not considering it “important enough” to check. Some respondents may have only checked terms that were of the highest importance to them and, thus, rated only those terms. Inherent biases such as not checking and subsequently not rating a term in RATA because the person did not notice the term can occur [4]. That is impossible in a forced testing method such as RATING. If the consumer was rushed, used a small screen, or simply missed a line of print for example, they could unintentionally not check some terms that otherwise might have “applied”. Primacy bias (checking those terms that occur earlier more often than those that occur later) among RATA “apply” responses also can occur even though terms were randomized across the respondents. However, it is possible that this bias could have influenced the total percentage of “apply” responses over all RATA respondents, even though the effect should be small.

It is important to note that the percentage of RATA “apply” responses for all motivation constructs for all food categories in all countries remained the same or reduced slightly when compared to corresponding CATA data. Prior studies [3,14] that compared CATA and RATA data showed that the percentage of “apply” responses for the attributes increased with use of RATA as compared to CATA. There are two possible explanations for this occurrence. Firstly, in those studies [3,14], one group of respondents saw the CATA question format of the survey while the other saw the RATA format, whereas, in the current study, respondents saw only the RATA question and we derived the CATA “apply” responses based on the first task in the RATA format. This implies that the CATA percentages shown here are part and parcel of the RATA data. Secondly, in some, but not all RATA question formats [3,14], consumers were asked to check terms that applied and then rate those that they had selected on the same page. It is possible that consumers who see the check box and the rating box on the same page are more likely to select “apply” more often. It is possible that the percentage of “apply” responses for RATA responses in the present study was lower because the consumers did not see the rating scale for RATA until after they had checked the “apply” response. However, if that were the case we might have seen increases in “apply” ratings for foods evaluated after the first one since respondents would have learned they would be asked to rate those that they checked as “apply”. We did not find that scenario. Regardless of such findings, we note that the focus of the current study were comparisons between RATA and RATING data and not CATA data.

We also found a few cases where RATA respondents changed their minds about the applicability of some motivation terms that they had previously checked as “apply” and instead rated them as “not at all important” or “not apply” for particular foods. This explains the change in percentages of “apply” responses between CATA and RATA (Table 2, Appendix A Table A1, Table A2, Table A3 and Table A4). For example, in Spain, while 35% of consumers marked the three subscales for the habits construct as an important motivation for them eating starch-rich foods (CATA), 2% of the same consumers changed their minds and rated it as “not apply” or “not at all important” in the rating portion of the RATA format. Thus, the resulting 33% “apply” responses for RATA. This shows that with the RATA question format, respondents took some time to think about their previous choices as they rated the selected terms for applicability for the particular food items something that the CATA question format does not provide for [14]. Nonetheless, just as Vidal et al. [3] stated, the small differences between RATA and CATA that were identified were particular to terms or attributes and food groups.

### 3.2. Ratios of RATING to RATA and Standard Indices for RATA and RATING

The fact that the ratio of “apply” responses of RATING to RATA question formats for all 16 constructs was greater than one reiterated our findings that the RATING question format had a higher percentage of “apply” responses as compared to the corresponding RATA data (Table 3, Appendix A Table A5, Table A6, Table A7 and Table A8). In addition, it was also evident that the importance of eating motivation constructs based on ratios of RATING to RATA varied among the five food groups depending on country. For example, in India, the importance of convenience in the eating of fruits and vegetables (Table A5) increased 12-fold using RATING, whereas it increased approximately only 6-fold for the starch-rich foods (Table A6). For the dairy category (Table 3), it increased 10-fold while for both protein-rich (Table A7) and desserts categories (Table A8) it increased 9-fold when the RATING question format was used in India. On the other hand, in China, importance for the convenience construct increased 23-fold for both protein-rich and desserts categories when the RATING survey format was used. Except for the liking motivation, similar variations were also noted for the other motivation constructs among different food groups across the five countries depending on whether RATA or RATING questionnaires were used.

We did notice, however, that several of the larger differences (30+) in construct importance between RATA and RATING data occurred among motivation constructs that received the lowest ratings overall. Such motivations included; affect regulation, social image, and social norms. It is also worth noting that for food groups such as protein-rich foods, dairy and fruits and vegetables, RATA respondents in Brazil did not consider (neither checked nor rated) any of the three terms or subscales for the affect regulation construct to be important motivations for eating the aforementioned foods (Table A1, Table A2 and Table A3). Consequently, the affect regulation construct received zero “apply” responses and zero values for corresponding standard indices of importance for the RATA question format (Table 3 and Table A5 and Table A7).

Overall the liking motivation construct had a higher percentage of “apply” responses for eating foods from the five food groups across all five countries (Table 4 and Appendix A Table A1, Table A2, Table A3 and Table A4). It did not matter what question format (whether RATING or RATA) was used, liking was the most important motivation for the consumers. These findings support several earlier studies that found a similar concept [1,49,56].

In consideration of that finding, the authors established standard indices of importance (SII) to identify how the other constructs compared with liking, the greatest motivation construct. A motivation construct found to have closely similar SII values for RATA and RATING would indicate that the relative importance accorded to it by either RATA or RATING were also closely similar [1]. For example, in Brazil, the difference between the two indices (SII:RATING minus SII:RATA) for the habits construct for starch-rich foods (0.05), protein-rich foods (0.2) and the dairy food category (0.26) could be explained as expected random variation among these values. However, the same cannot be said for the corresponding difference for the fruit and vegetables category (0.53) and dessert/sweet food category (0.71) in the same country. Clearly, in this case, consumers interpreted, processed and answered the RATING and RATA questions differently. Similar large differences (SII:RATING minus SII:RATA) were observed for other motivation constructs among the five food groups across all five countries. Further, the differences (SII:RATING minus SII:RATA) in the SII index ranged from 0.28 for the USA protein-rich foods category, to 0.95 for that same category in India. At this point, we found that not only was one survey format providing a significantly higher percentage of “apply” responses but also that the “apply” responses could be different. This strengthens the case for different information being provided by the two question formats which could result in variations in data analysis, interpretation and study conclusions by researchers.

To correctly understand the perceptions, opinions, beliefs and attitudes of consumers, researchers (e.g., sensory scientists, product developers, nutritionists, and marketers) should ask the right questions and, even more importantly, survey questions should be asked in a structure that collects the most accurate responses. Thus, determining what question format to use would be a critical step in the design process for upcoming online consumer studies. For the current study, we did not conduct exit interviews or focus groups (qualitative research studies) with the respondents (both RATA and RATING) from the five countries to validate the respective collected data for accuracy. As such, we could not prove that one survey format underestimated or overestimated the consumers’ responses. Further research in the validation of RATING and RATA data is warranted. It must be noted, however, that RATING has been the de facto standard for collecting sensory and consumer behavior data for decades. Although that does not mean that it is, in fact, correct, it does suggest that it is incumbent on authors proposing new methods, such as RATA, to show that the data produced are either similar or better than existing methods.

### 3.3. Comparison of Mean Scores for Eating Motivation Constructs

Results showed that mean scores for RATA and RATING data were similar for some attributes (constructs) for some countries and for some food categories. A case in point, both RATA and RATING survey respondents in China and the USA identified the habits eating motivation as very important to their eating of protein-rich foods (Table 4), dairy foods (Table A9) and Fruits and vegetables (Table A10). This could imply that in some situations particular respondents (or, in this case, respondents in certain countries) interpreted and processed the subscales or terms for particular constructs similarly for both RATA and RATING survey questions. Put simply, the same level of importance was placed on attributes/constructs in such cases. However, that was not always true. For example, although consumers in the US gave the same degree of importance (moderately important) to the habit construct for starch-rich foods using either format (Table A11), for dessert foods RATA respondents reported habits to be “very important” while corresponding RATING respondents found it to be “moderately important” (Table A12).

Except for China, where seven, eight and ten constructs were found to have similar mean scores for the two question formats for the protein-rich, dairy, and dessert food categories, respectively, other countries each had at most only five out of the 16 constructs that had similar mean scores for the two question formats for all food categories. RATA respondents from all countries pointed out that social image was a very important motivation for them eating protein-rich foods. However, corresponding RATING data for consumers in Western societies (Brazil, Spain, and the USA) identified the same construct as slightly important RATING data for China and India categorized social image as “moderately important”. Obviously, it would be illuminating to compare the impact of consumers’ demographic aspects on the RATA and RATING “apply” responses. However, this was not the objective for this paper.

Consumers’ RATA mean scores in Brazil, India, Spain and the USA for close to three-quarters (11/16) of the constructs for all food categories were significantly higher (greater level of importance) than those of corresponding RATING scores. In fact, in Spain, only two motivation constructs had similar mean scores for RATA and RATING for any food categories. At least fourteen had significantly higher mean scores based on RATA questioning as compared to RATING in every food group. This shows that consumer insights gathered using the RATA and the RATING question formats in online survey may not necessarily be the same. We noted also that overall the mean score values for the RATA question format were higher than those of corresponding RATING data for all food groups in all countries. This was true for all constructs except for the habits and convenience constructs regardless of whether the differences were statistically significant or not. There are two explanations for this occurrence. Firstly, the RATA question format requires respondents to select only attributes that are important and then rate the selected terms for “applicability” or level of importance. It can be assumed that all the terms selected at this point do “apply” though they may apply at different levels of importance. The RATING question provides no “opt out” option for but rather asks consumers to rate all statements or terms based on a scale from 1 to 5, where 1 means not at all important. If that score is chosen, the construct mean will decrease. It would appear that for ratings used during RATA, consumers were more likely to choose higher scores for importance since they had already stated that the motivation terms or statements were applicable. Furthermore, the five-point scale that was used included a “not at all important” option, which gave RATA respondents an “out” in case they changed their mind (i.e., checked a term as “apply” but then rated it as “not at all”). Such responses were included in the analysis and were added in as 1, which could increase the mean score slightly for RATA data. The case was not the same for the RATING responses which were treated as is [24,25,26,57].

We also noted eight cases that were linked to the habits and convenience constructs for particular food categories in Brazil, China and the USA, where the mean score for RATING was slightly higher than the corresponding RATA value. However, of these eight cases, it was only the habits construct under the starch-rich food category in China, where the mean score for RATING significantly differed from that of RATA (Table A11).

It is also important to note that differences between mean scores for RATING and RATA were smaller for constructs that were most frequently used by respondents. In India, for example, the differences for frequently used motivations (e.g., liking (0.52), habits (0.32) and convenience (0.32)) for the protein-rich category were less than the corresponding differences for infrequently used motivations (e.g., social image (0.87), affect regulation (0.96), and weight control (0.89)). As demonstrated also by the ratio of RATING to RATA “apply” responses and standard indices for the two question formats (Table 5, Appendix A Table A5, Table A6, Table A7 and Table A8), this indicates that the level of importance is likely to vary more among less-frequent and moderately used attributes or motivation constructs than frequent ones depending on whether RATA or RATING was used within a food category within a country.

### 3.4. Level of Importance for Motivation Constructs

#### 3.4.1. Based on Percentages of “Apply” Responses

**Figure 1:**
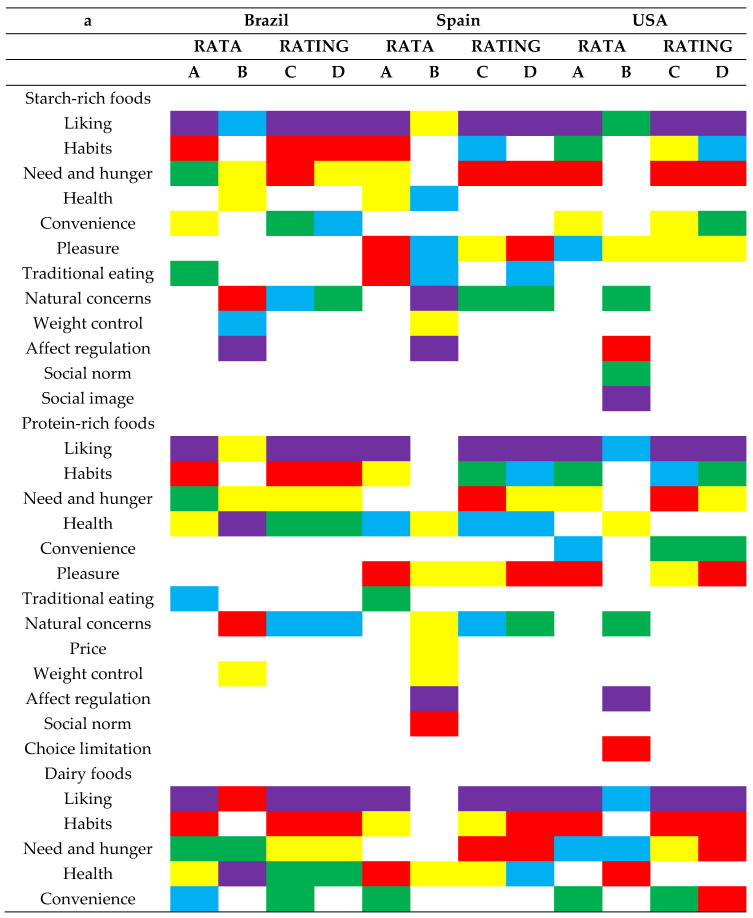

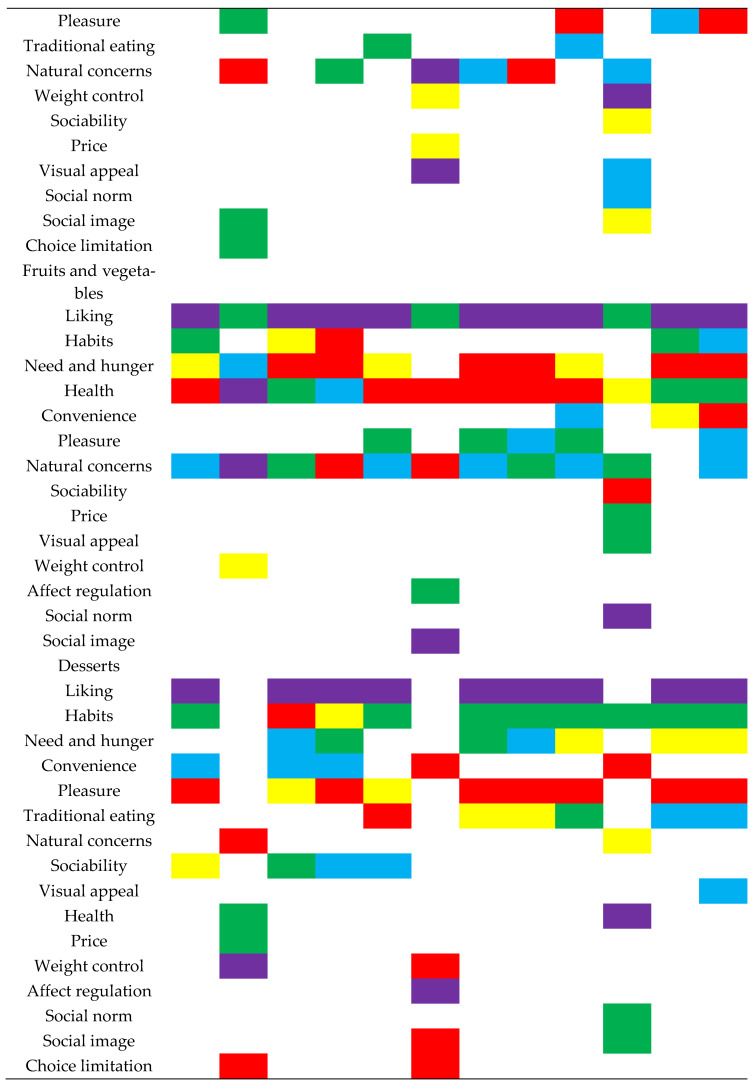

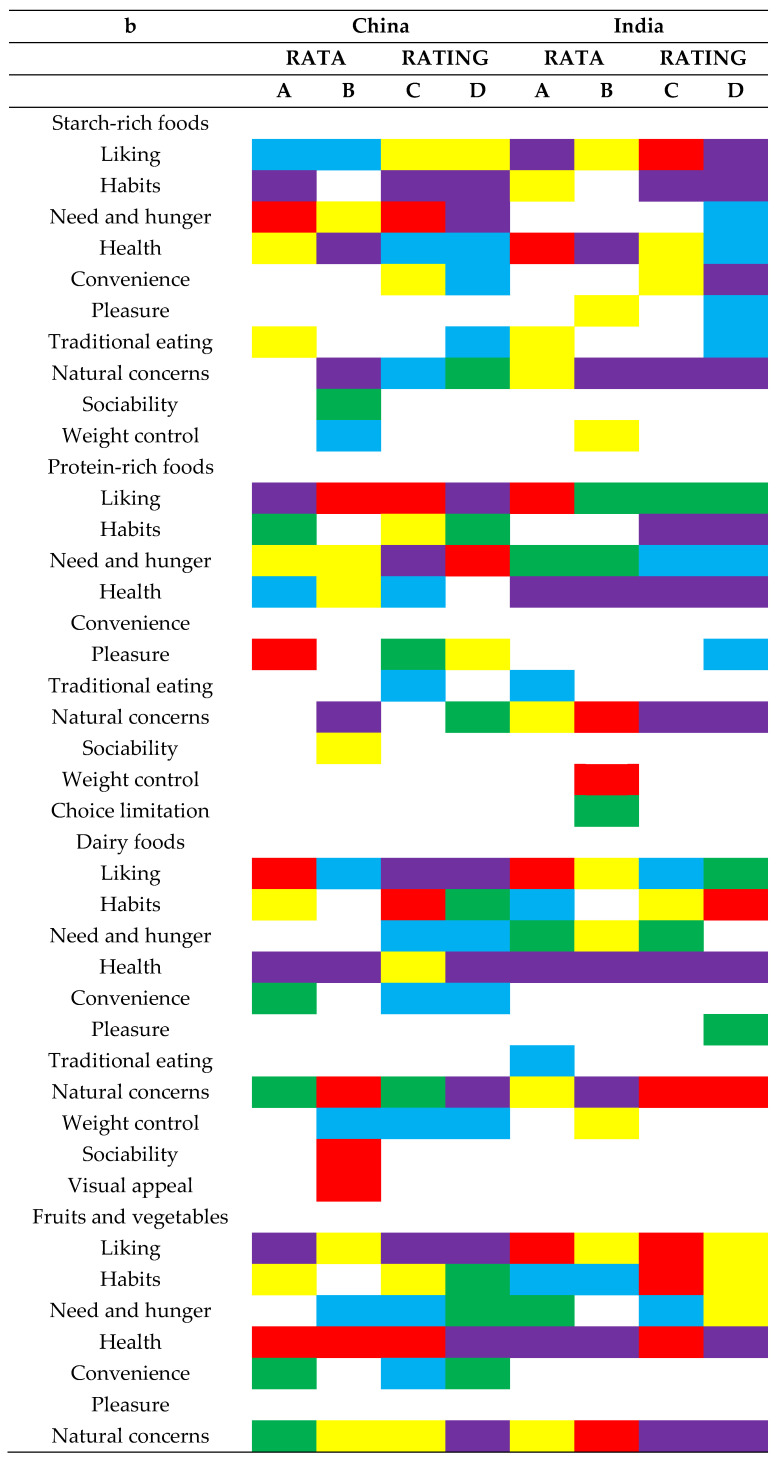

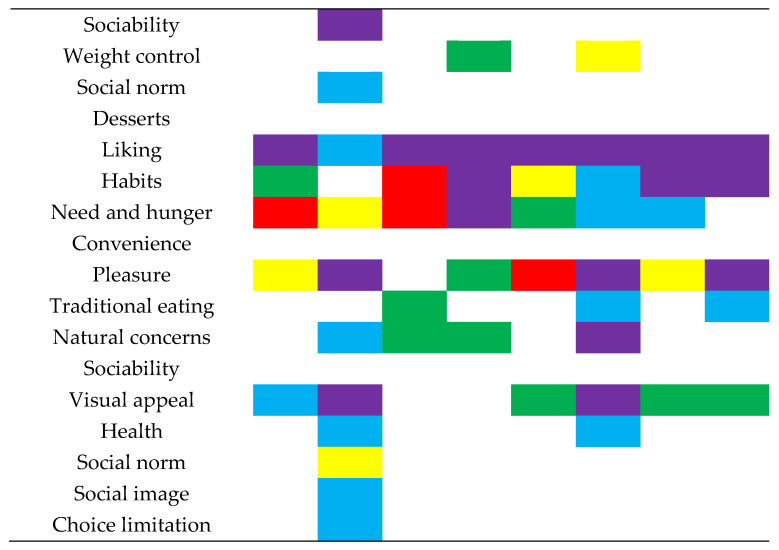
(**a**) Rank of the top five motivation constructs based on percentages of “apply” responses for RATA (**A**) and RATING (**C**) survey formats within each food group for Brazil, Spain, and the USA. Additionally, the top five motivation constructs based on mean scores per country for RATA (**B**) and RATING (**D**) within each food group for Brazil, Spain, and the USA are included. Rank color codes for the top five motivation constructs: purple = first position, red = second position, yellow = third position, green = fourth position, and blue = fifth position. (**b**) Rank of the top five motivation constructs based on percentages of “apply” responses for RATA (**A**) and RATING (**C**) survey formats within each food group for China and India. Additionally, the top five motivation constructs based on mean scores per country for RATA (**B**) and RATING (**D**) within each food group for China and India are included. Rank color codes for the top five motivation constructs: purple = first position, red = second position, yellow = third position, green = fourth position, and blue = fifth position.

Inspection of the relative positioning of the motivation constructs for RATA and RATING data for each country provided more understanding of the level of importance consumers accorded to each construct for the different food categories. Based on percentage of “apply” responses, consumers (both RATA and RATING respondents) in Western countries (Brazil, Spain, and the USA) identified the liking construct as the most important motivation for eating foods from all five categories (Figure 1a). This was not surprising since similar findings were attained by other authors in prior related studies [1,49,56].

However, for Asian countries (China and India), while the liking maintained the top most position for the food categories such as desserts, its ranking varied inconsistently for other food categories. Rate-All-That-Apply responses in China, for example, suggested that liking, pleasure, need and hunger were the leading drivers for the eating of protein-rich foods in that order, while RATING “apply” responses pointed out need and hunger, liking and habits in that order as the constructs that drove consumers in China to eat protein-rich foods. A similar case was seen in India where RATA data showed that consumers ate starch-rich foods mostly because they liked them while corresponding RATING data noted that Indians ate starch-rich foods mainly because it was a habit.

Eating motivations such as habits, need and hunger, convenience, and pleasure joined the liking construct and took positions among the top five constructs for eating behavior across most food categories and countries regardless of whether RATA or RATING survey question formats were used. Furthermore, traditional eating, natural concerns, health, weight control, and sociability were the other eating motivation constructs that also appeared among the top five positions, though these depended on the survey question format used, food category and country of target population. It is worth noting however that the level of importance for the latter set of constructs differed between RATA and RATING data more frequently as compared to the former set of motivation constructs. This further suggests that although some similarities between RATA and RATING data can be found, information collected using RATA questions and that collected using RATING questions may be different and may be interpreted differently potentially leading to different conclusions and decisions.

Another way to look at the ranking of attributes based on level of importance is to compare the data analysis approaches that were used for RATA and RATING data. To calculate the percentages of “apply” responses, RATA data were treated as CATA data, implying that the consumers’ responses were analyzed as binary numbers (1, 0) where a value of 1 was placed for each subscale or term that was selected as an important motivation for the consumption of a particular product category. Additionally, a value of 0 was placed for each subscale or term that was not selected as an important motivation for the consumption of a particular product category [12]. For RATA, the ratings or intensity scores were ignored except for cases where respondents changed their mind, i.e., the respondent checked a motivation term as “apply” but then rated it as “not at all important” (suggesting that they should not have checked the term to begin with), that specific data point was excluded from the analysis. Although the data show that rarely happened (<2% of cases), this decreased the percentage of apply responses and ranking slightly for RATA data. On the contrary, when computing the percentages of “apply” responses for RATING data, all response categories but “not at all important” were categorized as 1 and the only the “not at all important” were categorized as zero.

Additionally, those differences may depend on the particular product or sample (or food category) and country or culture of target population. Careful consideration is therefore recommended for consumer researchers when determining what question format to use in future online surveys for particular products because the level of importance given to each attribute or term may change depending on what survey format a respondent answers and the particular product(s) being assessed. More investigation into the accuracy of both methods may be needed before suggestions for use of one question format over the other can be made

#### 3.4.2. Based on Motivation Constructs’ Mean Scores

Across all five countries, the RATA question format gave a larger variety of top five motivation constructs based on mean scores as compared to those of the corresponding RATING format. This may be the result of differences in actual motivations across product categories that show up using the RATA format or could be an artifact of testing. We note that the RATING format produced more consistent top five motivation constructs when determined based on the percentage of “apply” responses and mean scores than did the RATA format. In RATA top five constructs sometimes changed depending on the data used.

In Brazil, except for convenience, which was replaced with natural concerns for the dairy food category, the same constructs were identified either based on percentages or mean scores for all five food categories using RATING. Another example was seen in Spain where the same key constructs were pinpointed based on percentages or mean scores for all food categories except for habits. Habits was replaced with traditional eating among the top five motivations for eating starch-rich foods when the mean scores for the constructs were compared using RATING data. That was not the case for the RATA survey format. We also noted that for the RATING survey format, the motivation construct ranking within the food categories did not change much particularly in Western countries (Brazil, Spain and the USA) and when the ranking did change, the constructs’ positions moved slightly. Conversely, for the RATA question format, several infrequently used motivation constructs such as affect regulation, social norms, social image, and choice limitation joined the list of top five constructs. For example, based on percentage of “apply” responses both RATA and RATING identified liking as the most important motivation for eating starch-rich foods. That was followed by habits. However, RATA mean scores suggested that affect regulation followed by natural concerns took the lead and liking came in fifth. Habits did not appear in the top five positions for motivations for eating starch-rich foods in Brazil. As for the RATING survey format, liking and habits both maintained their lead as key motivations for eating of starch-rich foods in Brazil. These findings suggest that ranking of attributes based on level of importance was more consistent for the RATING question format but changed significantly for RATA depending on whether ranking was based on percentages of “apply” responses or mean scores for attributes or constructs.

Meyners et al. [12] who analyzed RATA data both as CATA and also as a parametric found that RATA data were more meaningful when treated as parametric. At the time of writing, we did not find any research that provides more insights on how RATA and RATING compare in terms of discrimination among products, degree of importance or applicability of attributes. More investigation is needed to provide more understanding on ranking of attributes based on attribute percentage of “apply” responses and attribute mean scores.

### 3.5. Significant Differences among Samples

Overall, the total number of cases where the eating motivation constructs had significant differences among the food groups or samples for the RATING question format (*n* = 67) was higher than that of RATA (*n* = 20) (Table 5). This was showcased in all five countries. For example, in both Brazil and the USA, the RATA question format identified only four cases where significant differences were found among the samples, whereas, for RATING, the number of significant differences among samples was more than 3-fold higher (*n* = 14). Clearly, RATING was more discriminating among samples than RATA. This finding can be key for consumer researchers when designing future online surveys that would investigate characteristics of products or samples that are similar or closely related. Although it is not known which of the significant differences are “true”, the RATING format has been the gold standard for many decades and does appear to give somewhat different results than RATA. Further work is needed to determine impacts of such differences on findings in sensory and consumer behavior studies.

### 3.6. Comparison of Survey Format Completion Rates, Survey Mean Duration, Survey Liking, and Survey JAR Rating

#### 3.6.1. Consumers’ Survey Question Format Completion Rates

**Figure 2:**
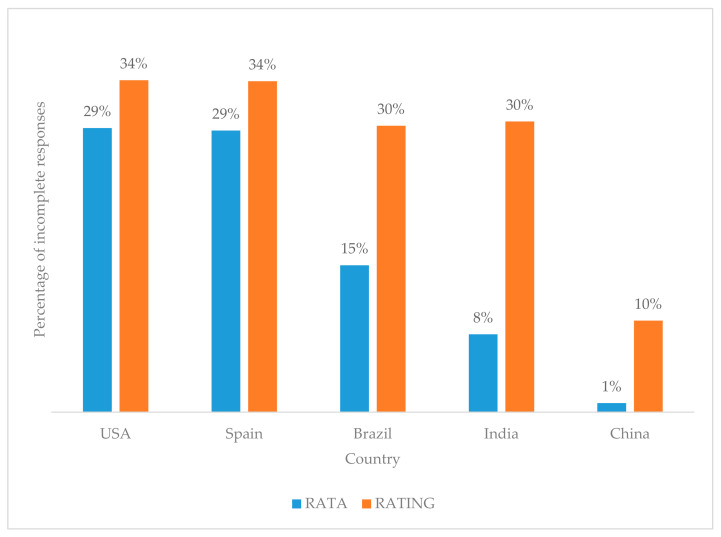
Percentage of incomplete responses for RATA and RATING per country. Incomplete questionnaires were not accepted or used and were not counted in the approximately 200 responses received per country per questionnaire type.

Chi-square tests showed that the percentage of incomplete responses for RATING data for countries such as Brazil, India and China were significantly higher than those of corresponding RATA data (Figure 2). This information could be beneficial when planning future international consumer studies (with RATA or RATING questions) in these five countries or countries with similar cultures.

#### 3.6.2. Consumers’ Survey Mean Duration

In China and Spain, consumers who answered the RATING format of the online survey took a significantly longer time to complete the survey compared to their counterparts who completed the RATA survey format (Table 6). That was not unexpected, especially since RATA respondents rated only those terms or attributes that they considered to “apply”, whereas RATING respondents rated all 47 terms. It was, however, surprising to note that in the USA, consumers took slightly longer (although not significantly) to complete the RATA questions than the RATING questions.

#### 3.6.3. Consumers’ Survey Just-about-Right (JAR) Rating

Apart from India, respondents from all five countries rated the RATING version of the survey as a little too long, while the RATA version of the survey was rated as JAR (Table 7). In India, however, the RATING survey format was rated as JAR, while the RATA format of the same survey was rated as a little too short. Except for the USA, this finding can be explained by the more time that respondents needed to complete the RATING format of the survey. These findings suggest that neither of the formats was overly burdensome to those who completed the questionnaire, but other factors such as survey liking and completion rates may be important.

#### 3.6.4. Consumers’ Survey Liking

In all five countries, the RATA versions of the survey were liked significantly more than the corresponding RATING versions of the same survey (Table 8). The higher liking gained by the RATA survey format could be expected by the JAR survey ratings. It must be noted that for both formats, the mean values for liking are positive, suggesting that at least for many consumers, the format they used in testing was acceptable.

In the case that the information collected from the shorter surveys satisfies the research objectives, then there may be no need to conduct longer surveys, especially since longer surveys would cost more. On the other hand, longer surveys could be used in place of shorter surveys in cases when more robust information is needed from the consumers. Additionally, longer surveys could negatively affect the online survey completion rates, which could increase the difficulty in attaining the required number of complete responses. However, survey duration and completion rates should not be used as a key basis for determining what question format to use in online survey questionnaires considering that quality of data could be impacted.

## 4. Study Limitations

It is possible that a proportion of the target population did not participate in this online survey (coverage error) simply because they lacked access to a stable and steady internet connection [58,59]. This implies a limitation to the inferences that can be made based on the current internet survey. According to Armstrong et al. [60], differences in response data, particularly in multi-country online surveys, can be ascribed in part to different recruitment software. Although we used the same software in all countries, the actual devices used (i.e., computer, phone, etc.) are likely different from country to country and may have some impact on the results. Similarly, paper ballots could be included in future survey designs as an option for respondents within the target populations who may not have access to the internet. Other survey limitations such as selection of particular samples (food groups) have been discussed previously [1].

## 5. Conclusions

This online survey showed that the RATING question format provided more “apply” responses for each attribute than the RATA question format. Additionally, based on the standard indices for RATA and RATING, the RATING question format showed better discrimination ability among attributes for all food categories in all countries as compared to the corresponding RATA data. Additionally, overall, the RATA mean scores for the attributes were found to be significantly higher (greater level of importance) than those of the RATING survey format. Further, the RATING question format showed better discrimination ability among food categories or samples than RATA for all motivation constructs or attributes and within all countries. More investigation into the use of the RATA and RATING question formats in future consumer research is needed.

## Figures and Tables

**Table 1:** Overview of demographic segmentation of respondents who completed the RATA and RATING question formats of the EMS in all five countries ^1^.

	Brazil		China		India		Spain		USA	
	A	B	A	B	A	B	A	B	A	B
Gender										
Males	100	115	103	100	128	136	106	107	106	105
Females	100	100	107	100	122	137	107	107	102	108
Age Group										
Boomers	50	65	51	49	54	57	52	52	54	54
Generation X	50	50	53	51	67	80	54	54	53	53
Millennials	50	50	52	50	56	62	54	54	53	53
Generation Z	50	50	54	50	73	74	53	54	48	53
Education Level										
Primary school or less	5	5	4	9	10	14	5	2	7	5
High school	96	89	86	68	35	55	111	93	101	86
College or university	99	121	120	123	205	204	97	119	100	122
Adults in Household										
One	10	13	7	2	12	14	1	0	47	56
Two or more	190	202	203	198	238	259	212	214	161	157
Children										
None	107	103	85	79	96	106	115	101	119	132
One or more	93	112	125	121	154	167	98	113	89	81

^1^ Number of respondents. A = RATA; B = RATING.

**Table 2:** Percentage of “apply” responses for CATA, RATA, and RATING for all five countries for the respective starch-rich foods.

	Brazil			China			India			Spain			USA		
	C	D	E	C	D	E	C	D	E	C	D	E	C	D	E
Liking	50	50	97	18	18	96	31	31	95	67	67	97	50	49	97
Habits	48	47	94	30	30	98	25	24	96	35	33	86	23	22	88
Need/Hunger	24	24	94	21	21	97	20	19	93	21	21	95	26	26	91
Health	23	23	85	19	19	95	26	26	94	22	22	84	14	14	79
Convenience	31	31	90	14	14	96	20	20	94	12	11	76	25	24	88
Pleasure	10	10	80	6	6	81	22	22	91	33	33	93	21	21	88
Trad. eating	25	24	68	20	19	92	24	24	92	32	32	85	16	15	72
Nat. concern	14	14	87	11	11	95	24	24	96	14	14	87	10	10	81
Sociability	6	6	68	5	5	84	11	11	85	16	16	82	2	2	53
Price	13	13	75	7	6	78	9	8	78	3	3	66	10	9	81
Visual app	4	4	54	4	3	80	16	16	85	10	10	69	5	5	68
Wt. control	13	13	80	6	6	87	15	15	89	6	6	73	10	10	71
Affect regul.	0	0	26	2	2	53	5	4	65	0	0	36	1	1	40
Social norm	5	5	56	7	7	75	7	7	72	3	3	61	3	3	54
Social image	2	2	33	5	5	76	10	9	72	6	6	50	2	2	46
Choice	15	14	70	13	13	84	16	16	86	4	4	65	5	5	67

C = percentage of “apply” responses for CATA; D = percentage of “apply” responses for RATA; E = percentage of “apply” responses for RATING.

**Table 3:** Ratios of RATING “apply” responses to RATA “apply” responses (R) and standard indices of importance for RATING (S) and RATA (T) “apply” responses for each motivation construct to the liking motivation construct for dairy foods in all five countries.

	Brazil			China			India			Spain			USA		
	R	S	T	R	S	T	R	S	T	R	S	T	R	S	T
Liking	2.1	1.00	1.00	4.9	1.00	1.00	4.2	1.00	1.00	2.4	1.00	1.00	2.5	1.00	1.00
Habits	2.9	0.98	0.72	6.8	0.99	0.72	7.0	1.02	0.61	3.5	0.97	0.66	6.1	0.97	0.40
Need/Hunger	4.5	0.95	0.45	16.0	0.93	0.29	5.0	1.00	0.84	7.4	0.99	0.32	9.3	0.95	0.26
Health	3.6	0.92	0.54	4.3	0.97	1.13	3.7	1.04	1.17	3.3	0.97	0.71	10.7	0.80	0.19
Convenience	4.8	0.91	0.41	9.1	0.92	0.51	10.1	0.99	0.41	5.9	0.92	0.37	9.1	0.95	0.26
Pleasure	7.8	0.81	0.22	15.3	0.86	0.28	6.9	0.97	0.59	7.6	0.88	0.28	5.7	0.92	0.40
Trad. eating	4.0	0.70	0.38	12.0	0.88	0.36	6.4	0.96	0.63	5.5	0.86	0.38	8.3	0.80	0.24
Nat. concern	11.4	0.90	0.17	9.1	0.97	0.53	4.4	1.03	0.99	10.1	0.93	0.22	31.0	0.77	0.06
Sociability	32.7	0.63	0.04	35.8	0.82	0.11	12.9	0.83	0.27	36.3	0.61	0.04	21.6	0.63	0.07
Price	15.5	0.77	0.11	18.4	0.81	0.22	11.8	0.81	0.29	18.6	0.74	0.10	11.5	0.85	0.19
Visual app	42.6	0.55	0.03	17.7	0.85	0.24	9.7	0.89	0.39	21.8	0.64	0.07	23.8	0.75	0.08
Wt. control	12.5	0.83	0.14	10.1	0.93	0.45	7.7	0.96	0.52	15.8	0.86	0.13	39.3	0.71	0.05
Affect regul.	na	0.33	0.00	43.9	0.64	0.07	16.1	0.71	0.19	31.3	0.46	0.04	73.3	0.50	0.02
Social norm	18.1	0.58	0.07	17.8	0.78	0.22	15.1	0.78	0.22	13.7	0.63	0.11	37.9	0.60	0.04
Social image	28.7	0.37	0.03	21.0	0.79	0.19	12.1	0.76	0.26	23.4	0.49	0.05	18.9	0.55	0.07
Choice	8.6	0.72	0.18	15.9	0.85	0.26	9.3	0.91	0.41	9.6	0.79	0.20	17.3	0.76	0.11

R = ratio of RATING “apply” responses to RATA “apply” responses, S = standard index of RATING “apply” responses for each construct to liking, and T = standard index of RATA “apply” responses for each construct to liking. na = not applicable because none of the corresponding construct’s terms or subscales were checked.

**Table 4:** Mean scores ^1^ for RATA and RATING survey formats and *p*-values for the corresponding two-sample *t*-test for each motivation construct for protein-rich foods in all five countries.

	Brazil			China			India			Spain			USA		
	K	L	M	K	L	M	K	L	M	K	L	M	K	L	M
Liking	4.3	4.2	0.063	4.1	3.6	<0.0001 *	4.3	3.8	<0.0001 *	4.1	4.0	0.088	4.2	4.0	0.006 *
Habits	3.8	3.9	0.530	3.3	3.2	0.559	4.2	3.9	0.005 *	3.6	3.3	0.015 *	3.4	3.3	0.375
Need/Hunger	4.3	3.8	<0.0001 *	3.9	3.4	0.001 *	4.3	3.7	<0.0001 *	4.0	3.5	0.000 *	4.0	3.6	0.000 *
Health	4.5	3.6	<0.0001 *	3.9	3.0	<0.0001 *	4.5	3.9	<0.0001 *	4.2	3.3	<0.0001 *	4.4	2.6	<0.0001 *
Convenience	3.8	3.1	0.000 *	3.2	2.8	0.222	4.0	3.6	0.030 *	4.0	3.2	<0.0001 *	3.6	3.3	0.053
Pleasure	4.0	3.1	<0.0001 *	3.7	3.3	0.005 *	4.2	3.7	0.000 *	4.2	3.7	<0.0001 *	4.0	3.7	0.002 *
Trad. eating	3.8	2.7	<0.0001 *	3.2	3.0	0.460	4.2	3.6	<0.0001 *	3.6	3.2	0.001 *	3.5	2.9	0.000 *
Nat. concern	4.4	3.5	<0.0001 *	4.2	3.2	0.009 *	4.4	3.9	<0.0001 *	4.2	3.4	<0.0001 *	4.3	2.7	<0.0001 *
Sociability	4.1	2.6	<0.0001 *	3.9	3.0	0.003 *	4.1	3.2	0.000 *	4.0	2.8	<0.0001 *	4.0	2.5	<0.0001 *
Price	4.1	2.8	<0.0001 *	3.2	2.7	0.225	4.0	3.0	<0.0001 *	4.2	2.5	<0.0001 *	3.9	2.9	<0.0001 *
Visual app	3.9	2.3	<0.0001 *	3.8	3.0	0.002 *	4.0	3.4	0.001 *	3.7	2.7	<0.0001 *	3.6	2.8	0.009 *
Wt. control	4.3	3.0	<0.0001 *	3.7	2.7	0.008 *	4.4	3.5	<0.0001 *	4.2	2.7	<0.0001 *	3.8	2.4	0.006 *
Affect regul.	1.0	1.6	0.593	3.1	2.5	0.206	3.7	2.7	0.004 *	4.6	2.0	<0.0001 *	4.8	2.1	<0.0001 *
Social norm	3.8	2.5	<0.0001 *	3.5	2.8	0.062	4.1	2.9	<0.0001 *	4.5	2.4	<0.0001 *	4.0	2.4	0.001 *
Social image	4.0	1.8	<0.0001 *	3.7	2.8	0.009 *	3.9	3.0	0.001 *	4.1	2.3	<0.0001 *	3.9	2.2	<0.0001 *
Choice	4.0	2.9	<0.0001 *	3.4	2.9	0.231	4.3	3.4	<0.0001 *	4.1	2.8	<0.0001 *	4.7	2.6	<0.0001 *

K = mean scores for RATA, L = mean scores for RATING, and M = *p*-values for two-sample *t*-test. ^1^ Five-point scale: 1 = not at all important, 2 = slightly important, 3 = moderately important, 4 = very important, and 5 = extremely important. * *p*-values were lower than the significance level alpha = 0.05, implying that particular mean scores for RATA and RATING significantly differed.

**Table 5:** *p*-values of analysis of variance (ANOVA) for food categories or samples for RATA (A) and RATING (B) data for all eating motivation constructs.

	Brazil		China		India		Spain		USA		TSD	
	A	B	A	B	A	B	A	B	A	B	A	B
Liking	0.339	<0.0001 *	0.017 *	0.049 *	0.271	0.100	0.372	0.000 *	0.259	<0.0001 *	1	4
Habits	0.020 *	<0.0001 *	0.354	<0.0001 *	0.052	0.001 *	<0.0001 *	<0.0001 *	<0.0001 *	0.084	3	4
Need/Hunger	0.905	<0.0001 *	0.320	<0.0001 *	0.249	<0.0001 *	0.606	<0.0001 *	0.353	<0.0001 *	0	5
Health	0.004 *	<0.0001 *	0.732	<0.0001 *	0.000 *	<0.0001 *	0.003 *	<0.0001 *	0.013 *	<0.0001 *	4	5
Convenience	0.150	<0.0001 *	0.392	<0.0001 *	0.032*	<0.0001 *	0.034 *	<0.0001 *	0.026 *	<0.0001 *	3	5
Pleasure	0.183	<0.0001 *	0.002 *	0.001 *	0.791	0.017 *	0.032 *	<0.0001 *	0.172	<0.0001 *	2	5
Trad.eating	0.016 *	0.713	0.295	<0.0001 *	0.590	0.008 *	0.016 *	<0.0001 *	0.167	0.004 *	2	4
Nat.concern	0.749	<0.0001 *	0.694	<0.0001 *	0.269	<0.0001 *	0.904	<0.0001 *	<0.0001 *	<0.0001 *	1	5
Sociability	0.863	<0.0001 *	0.549	0.067	0.833	0.009 *	0.313	<0.0001 *	0.542	0.000 *	0	4
Price	0.792	<0.0001 *	0.320	0.036 *	0.914	0.423	0.529	<0.0001 *	0.892	0.000 *	0	4
Visual app	0.612	<0.0001 *	0.299	0.435	0.788	0.036 *	0.018 *	0.013 *	0.076	0.009 *	1	4
Wt.control	0.081	<0.0001 *	0.995	<0.0001 *	0.020 *	<0.0001 *	0.918	<0.0001 *	0.513	<0.0001 *	1	5
Affect regul.	0.000 *	<0.0001 *	0.111	<0.0001 *	0.384	0.000 *	0.657	0.000 *	0.352	<0.0001 *	1	5
Social norms	0.776	0.085	0.050 *	0.016 *	0.061	0.054	0.151	0.397	0.834	0.021 *	1	2
Social image	0.063	0.012 *	0.507	0.154	0.805	0.026 *	0.258	0.112	0.663	0.003 *	0	3
Choice limit	0.202	0.032 *	0.720	0.043 *	0.229	0.093	0.840	<0.0001 *	0.127	0.120	0	3
TSD	4	14	3	13	3	12	6	14	4	14	20	67

A = RATA, B = RATING, and TSD = Total Significant Differences. * *p*-values were lower than the significance level alpha = 0.05, implying that particular mean scores among the food categories within a question format differed significantly.

**Table 6:** Means and standard deviations ^†^ and *p*-values for the survey mean duration for RATA and RATING per country.

	Brazil		China		India		Spain		USA	
	Means	SD	Means	SD	Means	SD	Means	SD	Means	SD
RATA	36.5	60.3	17.9	17.6	34.6	98.1	18.0	14	37.8	288.9
RATING	44.7	81.3	29.5	34.3	46.0	141	34.7	88.3	26.9	19.4
*p*-value	0.248		<0.0001 *		0.287		0.007 *		0.582	

^†^ Mean duration and standard deviations in minutes; * *p*-values with an asterisk indicate that RATA and RATING means differed significantly (*p* ≤ 0.05).

**Table 7:** Means and standard deviations ^†^ *p*-values for just-about-right ratings for RATA and RATING per country.

	Brazil		China		India		Spain		USA	
	Means	SD	Means	SD	Means	SD	Means	SD	Means	SD
RATA	4.4	0.7	4.3	0.8	3.1	1.8	4.5	0.8	4.4	1.0
RATING	5.0	1.1	5.0	1.2	3.6	2.1	5.2	1.1	5.0	1.4
*p*-value	<0.0001 *		<0.0001 *		0.007 *		<0.0001 *		<0.0001 *	

^†^ Seven-point scale: 1 = much too short, 2 = too short, 3 = a little too short, 4 = just about right (JAR), and 5 = a little too long, 6 = too long, and 7 = much too long; * *p*-values with an asterisk indicate that RATA and RATING means differed significantly (*p* ≤ 0.05).

**Table 8:** Means ^†^ and *p*-values for survey liking for RATA and RATING per country.

	Brazil		China		India		Spain		USA	
	Means	SD	Means	SD	Means	SD	Means	SD	Means	SD
RATA	4.3	0.7	4.0	0.8	4.4	0.8	4.1	0.7	4.0	0.9
RATING	3.9	1.0	3.5	1.1	4.1	1.0	3.8	0.9	3.5	1.1
*p*-value	<0.0001 *		<0.0001 *		0.002 *		<0.0001 *		<0.0001 *	

^†^ Five-point scale: 1 = I hated taking it, 2 = I did not like taking it, 3 = I have no feelings either way, 4 = I liked taking it and 5 = I liked it a lot; * *p*-values with an asterisk indicate that RATA and RATING means differed significantly (*p* ≤ 0.05).

## Data Availability

The data from this study are not publicly available.

## Appendix A

**Table A1:** Percentage of “apply” responses for CATA, RATA, and RATING for all five countries for the respective protein-rich foods.

	Brazil			China			India			Spain			USA		
	C	D	E	C	D	E	C	D	E	C	D	E	C	D	E
Liking	53	53	99	32	32	96	25	25	95	59	59	98	48	47	96
Habits	40	40	95	9	9	92	16	16	97	27	27	91	19	18	87
Need/Hunger	25	25	94	13	12	97	20	20	94	18	18	94	21	20	91
Health	29	29	88	7	7	86	34	34	97	19	19	89	5	5	67
Convenience	8	8	82	3	3	76	11	11	93	14	14	87	18	17	88
Pleasure	9	9	76	14	14	90	17	17	93	34	34	93	25	24	90
Trad. eating	19	19	67	6	6	86	18	18	91	20	20	86	10	10	76
Nat. concern	10	10	87	2	2	85	23	23	97	11	11	89	2	2	65
Sociability	3	3	67	3	3	84	6	6	81	6	6	79	5	4	65
Price	5	5	75	2	2	71	8	8	77	3	3	66	7	6	77
Visual app	2	2	54	5	5	84	10	9	85	7	7	70	4	4	73
Wt. control	10	10	78	3	3	75	13	13	88	7	7	76	1	1	59
Affect regul.	0	0	26	1	1	62	4	3	62	2	2	42	1	1	42
Social norm	6	6	59	3	3	78	6	5	70	2	2	63	2	2	55
Social image	1	1	33	4	3	78	5	5	71	4	4	54	4	4	47
Choice	13	13	72	3	3	79	14	14	84	6	6	75	3	3	66

C = percentage of “apply” responses for CATA, D = percentage of “apply” responses for RATA, and E = percentage of “apply” responses for RATING.

**Table A2:** Percentage of “apply” responses for CATA, RATA, and RATING for all five countries for the respective milk and dairy foods.

	Brazil			China			India			Spain			USA		
	C	D	E	C	D	E	C	D	E	C	D	E	C	D	E
Liking	45	45	96	20	20	97	23	23	94	39	39	94	37	37	94
Habits	33	33	94	15	14	96	14	14	96	26	26	91	16	15	91
Need/Hunger	20	20	91	6	6	90	19	19	95	13	13	93	9	9	89
Health	24	24	88	22	22	95	26	26	98	28	28	91	7	7	75
Convenience	18	18	88	10	10	90	9	9	93	15	15	86	10	10	88
Pleasure	10	10	78	5	5	83	13	13	92	11	11	83	15	15	86
Trad. eating	18	17	67	7	7	85	14	14	90	15	15	81	10	9	75
Nat. concern	8	8	87	11	10	94	22	22	97	9	9	88	2	2	72
Sociability	2	2	61	2	2	80	7	6	79	2	2	57	3	3	59
Price	5	5	74	5	4	78	7	6	76	4	4	70	7	7	80
Visual app	1	1	53	5	5	82	9	9	84	3	3	60	3	3	70
Wt. control	6	6	80	9	9	90	12	12	90	5	5	81	2	2	66
Affect regul.	0	0	32	2	2	62	5	4	67	1	1	43	1	1	46
Social norm	3	3	56	4	4	76	6	5	74	4	4	60	1	1	56
Social image	1	1	36	4	4	77	6	6	72	2	2	46	3	3	52
Choice	8	8	69	5	5	82	9	9	86	8	8	74	4	4	71

C = percentage of “apply” responses for CATA, D = percentage of “apply” responses for RATA, and E = percentage of “apply” responses for RATING.

**Table A3:** Percentage of “apply” responses for CATA, RATA, and RATING for all five countries for the respective fruits and vegetables.

	Brazil			China			India			Spain			USA		
	C	D	E	C	D	E	C	D	E	C	D	E	C	D	E
Liking	51	51	98	24	24	96	26	25	95	43	43	97	37	37	96
Habits	22	22	93	14	14	94	15	15	95	15	14	90	12	11	88
Need/Hunger	29	29	94	7	7	92	17	16	93	26	25	95	21	21	95
Health	35	35	90	19	19	95	26	26	95	34	34	95	27	27	88
Convenience	10	10	83	11	11	92	7	7	86	12	12	88	12	12	91
Pleasure	10	10	77	5	5	85	13	12	91	17	17	92	16	16	85
Trad. eating	11	11	61	5	5	82	13	12	87	6	6	76	6	5	64
Nat. concern	18	18	90	11	11	94	22	22	96	16	16	91	12	12	83
Sociability	1	1	52	3	3	80	5	5	77	2	2	51	1	1	52
Price	9	9	75	5	5	78	12	11	79	5	5	71	7	7	81
Visual app	2	2	51	4	4	85	9	9	80	4	4	66	4	4	65
Wt. control	17	17	80	8	8	90	13	13	89	12	12	81	11	11	83
Affect regul.	0	0	32	1	1	63	4	3	68	1	1	44	1	1	46
Social norm	5	5	55	4	4	78	5	4	71	2	2	60	2	2	56
Social image	1	1	33	3	3	79	7	7	72	2	2	45	2	2	48
Choice	7	7	67	3	3	84	9	8	83	4	4	77	4	4	71

C = percentage of “apply” responses for CATA, D = percentage of “apply” responses for RATA, and E = percentage of “apply” responses for RATING.

**Table A4:** Percentage of “apply” responses for CATA, RATA, and RATING for all five countries for the respective desserts.

	Brazil			China			India			Spain			USA		
	C	D	E	C	D	E	C	D	E	C	D	E	C	D	E
Liking	56	56	96	21	21	95	30	30	92	33	33	95	36	35	95
Habits	11	11	88	8	8	94	14	13	92	9	9	87	7	6	83
Need/Hunger	7	7	76	12	12	94	13	12	86	6	6	87	8	7	86
Health	2	2	56	6	6	87	8	7	80	2	2	67	2	2	58
Convenience	10	10	76	4	4	89	9	9	81	3	3	78	2	2	75
Pleasure	17	16	81	9	9	87	17	16	91	22	22	94	30	29	92
Trad. eating	9	9	69	6	6	91	10	9	85	31	31	89	7	6	81
Nat. concern	2	2	54	3	3	91	7	7	80	2	2	76	2	2	63
Sociability	12	12	77	5	5	85	9	8	84	7	7	80	4	4	70
Price	2	2	67	6	6	83	6	6	75	1	1	63	3	3	67
Visual app	5	5	69	7	7	86	13	12	88	4	4	73	4	4	74
Wt. control	1	1	48	5	5	85	7	7	77	1	1	55	1	1	57
Affect regul.	6	6	55	4	4	80	5	4	72	1	1	51	1	1	58
Social norm	0	0	53	3	3	84	5	4	74	3	3	65	2	2	63
Social image	1	1	43	5	5	81	6	5	79	2	2	52	3	3	58
Choice	3	3	63	5	5	88	6	5	80	1	1	64	5	4	66

C = percentage of “apply” responses for CATA, D = percentage of “apply” responses for RATA, and E = percentage of “apply” responses for RATING.

**Table A5:** Ratios of RATING “apply” responses to RATA “apply” responses (R) and standard indices of importance for RATING (S) and RATA (T) “apply” responses for each motivation construct to the liking motivation construct for fruits and vegetables in all five countries.

	Brazil			China			India			Spain			USA		
	R	S	T	R	S	T	R	S	T	R	S	T	R	S	T
Liking	1.9	1.00	1.00	4.0	1.00	1.00	3.7	1.00	1.00	2.3	1.00	1.00	2.6	1.00	1.00
Habits	4.3	0.95	0.42	6.9	0.97	0.57	6.4	1.00	0.58	6.5	0.93	0.33	7.6	0.91	0.31
Need/Hunger	3.2	0.96	0.58	12.7	0.95	0.30	5.7	0.98	0.64	3.7	0.97	0.60	4.6	0.99	0.56
Health	2.6	0.92	0.69	5.0	0.98	0.79	3.6	1.00	1.03	2.8	0.97	0.79	3.3	0.91	0.72
Convenience	8.7	0.85	0.19	8.5	0.96	0.45	12.3	0.90	0.27	7.5	0.90	0.27	7.8	0.94	0.32
Pleasure	8.0	0.79	0.19	16.9	0.88	0.21	7.4	0.96	0.49	5.3	0.95	0.41	5.5	0.88	0.42
Trad. eating	5.8	0.62	0.21	17.7	0.85	0.20	7.2	0.92	0.48	13.5	0.79	0.13	12.0	0.67	0.15
Nat. concern	5.0	0.92	0.36	8.8	0.97	0.45	4.4	1.01	0.87	5.6	0.94	0.38	6.7	0.86	0.34
Sociability	95.5	0.53	0.01	30.7	0.83	0.11	15.4	0.81	0.20	24.5	0.52	0.05	54.5	0.54	0.03
Price	8.9	0.77	0.17	16.8	0.81	0.20	7.1	0.83	0.44	15.0	0.73	0.11	12.4	0.84	0.18
Visual app	31.5	0.52	0.03	24.0	0.88	0.15	9.1	0.84	0.35	16.7	0.68	0.09	17.7	0.67	0.10
Wt. control	4.7	0.82	0.34	11.2	0.93	0.34	7.0	0.94	0.50	6.8	0.83	0.28	7.3	0.86	0.31
Affect regul.	na	0.32	0.00	34.0	0.66	0.08	23.7	0.72	0.11	46.4	0.45	0.02	59.6	0.47	0.02
Social norm	10.8	0.56	0.10	18.2	0.81	0.18	17.3	0.75	0.16	24.4	0.61	0.06	26.5	0.58	0.06
Social image	60.3	0.33	0.01	26.5	0.82	0.13	11.0	0.75	0.26	29.6	0.46	0.04	19.3	0.50	0.07
Choice	9.9	0.68	0.13	30.0	0.87	0.12	9.9	0.88	0.33	19.4	0.79	0.09	16.4	0.73	0.12

R = ratio of RATING “apply” responses to RATA “apply” responses, S = standard index of RATING “apply” responses for each construct to liking, and T = standard index of RATA “apply” responses for each construct to liking. na = not applicable because none of the corresponding construct’s terms or subscales were checked.

**Table A6:** Ratios of RATING “apply” responses to RATA “apply” responses (R) and standard indices of importance for RATING (S) and RATA (T) “apply” responses for each motivation construct to the liking motivation construct for starch-rich foods in all five countries.

	Brazil			China			India			Spain			USA		
	R	S	T	R	S	T	R	S	T	R	S	T	R	S	T
Liking	1.9	1.00	1.00	5.4	1.00	1.00	3.0	1.00	1.00	1.4	1.00	1.00	2.0	1.00	1.00
Habits	2.0	0.98	0.93	3.3	1.02	1.67	4.0	1.01	0.78	2.6	0.88	0.49	4.1	0.91	0.44
Need/Hunger	3.9	0.98	0.48	4.6	1.01	1.18	4.9	0.98	0.61	4.4	0.97	0.32	3.6	0.94	0.52
Health	3.7	0.88	0.47	4.9	0.98	1.07	3.6	0.99	0.83	3.8	0.87	0.33	5.7	0.81	0.28
Convenience	2.9	0.93	0.61	7.0	0.99	0.76	4.6	0.99	0.65	6.7	0.79	0.17	3.6	0.91	0.50
Pleasure	7.7	0.83	0.21	12.9	0.84	0.35	4.1	0.96	0.71	2.8	0.96	0.49	4.2	0.91	0.43
Trad. eating	2.9	0.70	0.47	4.8	0.95	1.06	3.9	0.96	0.76	2.6	0.88	0.49	5.0	0.74	0.30
Nat. concern	6.3	0.90	0.27	8.8	0.99	0.60	4.1	1.01	0.76	6.1	0.90	0.21	8.1	0.84	0.20
Sociability	10.8	0.70	0.13	16.9	0.87	0.28	8.1	0.90	0.34	5.2	0.84	0.23	21.7	0.54	0.05
Price	6.0	0.78	0.25	12.0	0.81	0.36	9.3	0.83	0.27	19.6	0.68	0.05	8.6	0.83	0.19
Visual app	13.8	0.56	0.08	22.9	0.83	0.19	5.3	0.89	0.51	6.8	0.71	0.15	15.1	0.70	0.09
Wt. control	6.4	0.83	0.25	13.8	0.91	0.35	6.0	0.94	0.47	11.3	0.75	0.10	7.0	0.73	0.20
Affect regul.	153.7	0.27	0.00	26.7	0.55	0.11	14.8	0.68	0.14	74.3	0.37	0.01	38.2	0.41	0.02
Social norm	10.4	0.58	0.11	11.0	0.78	0.38	10.9	0.75	0.21	22.2	0.62	0.04	18.3	0.56	0.06
Social image	17.9	0.35	0.04	15.2	0.79	0.28	8.0	0.76	0.29	7.4	0.51	0.10	18.9	0.47	0.05
Choice	4.8	0.72	0.29	6.4	0.88	0.74	5.4	0.91	0.51	17.9	0.66	0.05	14.3	0.69	0.10

R = ratio of RATING “apply” responses to RATA “apply” responses, S = standard index of RATING “apply” responses for each construct to liking, and T = standard index of RATA “apply” responses for each construct to liking. na = not applicable because none of the corresponding construct’s terms or subscales were checked.

**Table A7:** Ratios of RATING “apply” responses to RATA “apply” responses (R) and standard indices of importance for RATING (S) and RATA (T) “apply” responses for each motivation construct to the liking motivation construct for protein-rich foods in all five countries.

	Brazil			China			India			Spain			USA		
	R	S	T	R	S	T	R	S	T	R	S	T	R	S	T
Liking	1.9	1.00	1.00	3.0	1.00	1.00	3.7	1.00	1.00	1.7	1.00	1.00	2.0	1.00	1.00
Habits	2.4	0.96	0.76	10.4	0.95	0.28	6.2	1.02	0.62	3.4	0.93	0.46	4.9	0.91	0.38
Need/Hunger	3.7	0.95	0.48	7.7	1.01	0.39	4.6	0.99	0.80	5.4	0.96	0.30	4.6	0.95	0.42
Health	3.0	0.89	0.55	13.1	0.89	0.21	2.9	1.02	1.33	4.7	0.91	0.32	14.1	0.70	0.10
Convenience	10.1	0.83	0.15	23.2	0.79	0.10	8.9	0.98	0.41	6.4	0.89	0.23	5.2	0.92	0.36
Pleasure	8.4	0.77	0.17	6.4	0.94	0.44	5.5	0.98	0.66	2.7	0.95	0.58	3.8	0.94	0.51
Trad. eating	3.5	0.67	0.36	15.6	0.90	0.17	5.0	0.96	0.72	4.4	0.88	0.33	7.6	0.80	0.21
Nat. concern	8.4	0.87	0.20	41.8	0.89	0.06	4.2	1.02	0.91	7.8	0.91	0.19	26.4	0.68	0.05
Sociability	20.8	0.67	0.06	27.3	0.87	0.10	14.8	0.86	0.22	12.9	0.81	0.10	14.9	0.68	0.09
Price	15.7	0.76	0.09	43.4	0.74	0.05	9.8	0.81	0.31	21.6	0.68	0.05	11.9	0.81	0.14
Visual app	21.8	0.55	0.05	17.0	0.87	0.15	9.3	0.89	0.36	10.5	0.72	0.11	20.8	0.76	0.07
Wt. control	8.0	0.79	0.19	28.1	0.78	0.08	6.6	0.93	0.53	11.4	0.78	0.11	41.9	0.62	0.03
Affect regul.	na	0.27	0.00	60.2	0.64	0.00	18.6	0.65	0.13	27.7	0.43	0.03	40.0	0.44	0.02
Social norm	9.9	0.60	0.11	25.4	0.81	0.10	14.0	0.74	0.20	28.3	0.64	0.04	34.6	0.57	0.03
Social image	23.6	0.34	0.03	23.7	0.81	0.10	14.8	0.75	0.19	14.3	0.55	0.06	12.3	0.49	0.08
Choice	5.8	0.73	0.24	28.7	0.82	0.09	6.1	0.88	0.54	12.3	0.77	0.10	19.2	0.69	0.07

R = ratio of RATING “apply” responses to RATA “apply” responses, S = standard index of RATING “apply” responses for each construct to liking, and T = standard index of RATA “apply” responses for each construct to liking. na = not applicable because none of the corresponding construct’s terms or subscales were checked.

**Table A8:** Ratios of RATING “apply” responses to RATA “apply” responses (R) and standard indices of importance for RATING (S) and RATA (T) “apply” responses for each motivation construct to the liking motivation construct for dessert foods in all five countries.

	Brazil			China			India			Spain			USA		
	R	S	T	R	S	T	R	S	T	R	S	T	R	S	T
Liking	1.7	1.00	1.00	4.6	1.00	1.00	3.1	1.00	1.00	2.9	1.00	1.00	2.7	1.00	1.00
Habits	8.0	0.91	0.20	12.1	0.99	0.38	7.0	1.01	0.44	9.6	0.92	0.28	14.3	0.88	0.17
Need/Hunger	10.6	0.79	0.13	7.6	0.99	0.60	7.3	0.94	0.40	15.5	0.92	0.17	12.3	0.90	0.20
Health	33.4	0.58	0.03	14.1	0.92	0.30	11.3	0.87	0.24	27.3	0.71	0.07	27.5	0.61	0.06
Convenience	7.7	0.79	0.18	22.9	0.94	0.19	9.4	0.89	0.29	26.4	0.82	0.09	32.4	0.79	0.07
Pleasure	5.0	0.84	0.29	9.7	0.92	0.43	5.6	1.00	0.55	4.2	0.99	0.68	3.1	0.96	0.83
Trad. eating	7.6	0.72	0.16	15.7	0.96	0.28	9.5	0.93	0.30	2.9	0.95	0.93	12.8	0.85	0.18
Nat. concern	32.0	0.56	0.03	33.5	0.96	0.13	12.2	0.88	0.22	31.1	0.81	0.07	33.7	0.66	0.05
Sociability	6.7	0.80	0.21	16.8	0.89	0.25	10.9	0.92	0.26	10.9	0.84	0.22	16.7	0.74	0.12
Price	30.9	0.69	0.04	14.3	0.88	0.28	12.7	0.82	0.20	51.7	0.67	0.04	22.2	0.71	0.09
Visual app	13.7	0.72	0.09	13.0	0.91	0.32	7.0	0.96	0.42	16.5	0.77	0.13	18.7	0.78	0.11
Wt. control	67.0	0.50	0.01	15.6	0.89	0.26	11.7	0.84	0.22	75.2	0.59	0.02	61.6	0.60	0.03
Affect regul.	9.5	0.57	0.10	20.6	0.84	0.19	17.0	0.78	0.14	41.5	0.54	0.04	41.6	0.61	0.04
Social norm	222.0	0.55	0.00	27.0	0.88	0.15	16.8	0.80	0.15	18.9	0.69	0.10	30.0	0.66	0.06
Social image	44.5	0.44	0.02	15.0	0.86	0.26	15.2	0.86	0.18	30.1	0.55	0.05	17.7	0.61	0.09
Choice	21.8	0.65	0.05	18.9	0.93	0.23	15.2	0.88	0.18	43.3	0.67	0.04	17.3	0.70	0.11

R = ratio of RATING “apply” responses to RATA “apply” responses, S = standard index of RATING “apply” responses for each construct to liking, and T = standard index of RATA “apply” responses for each construct to liking. na = not applicable because none of the corresponding construct’s terms or subscales were checked.

**Table A9:** Mean scores1 for RATA and RATING survey formats and *p*-values for the corresponding two-sample *t*-test for each motivation construct for dairy foods in all five countries.

	Brazil			China			India			Spain			USA		
	K	L	M	K	L	M	K	L	M	K	L	M	K	L	M
Liking	4.4	4.1	0.001 *	3.8	3.6	0.098	4.3	3.9	<0.0001 *	4.1	3.8	0.000 *	4.1	3.7	<0.0001 *
Habits	3.9	3.8	0.533	3.6	3.5	0.585	4.2	4.0	0.098	3.6	3.4	0.121	3.2	3.4	0.433
Need/Hunger	4.3	3.7	<0.0001 *	3.6	3.3	0.283	4.3	3.8	0.05 *	4.1	3.4	<0.0001 *	4.1	3.4	0.000 *
Health	4.6	3.6	<0.0001 *	4.1	3.6	<0.0001 *	4.4	4.1	0.000 *	4.2	3.3	<0.0001 *	4.3	2.9	<0.0001 *
Convenience	4.1	3.5	0.000 *	3.6	3.3	0.084	4.0	3.8	0.108	3.9	3.2	<0.0001 *	3.6	3.4	0.165
Pleasure	4.3	3.2	<0.0001 *	3.7	3.2	0.035 *	4.2	3.9	0.036 *	3.9	3.2	0.000 *	4.0	3.4	0.000 *
Trad. eating	3.9	2.8	<0.0001 *	3.7	3.1	0.007 *	4.1	3.7	0.005 *	3.8	3.0	<0.0001 *	3.3	2.9	0.074
Nat. concern	4.4	3.6	0.000 *	3.9	3.6	0.053	4.4	4.0	0.000 *	4.3	3.4	<0.0001 *	4.1	2.9	0.009 *
Sociability	4.1	2.5	0.002 *	3.9	2.9	0.012 *	3.9	3.2	0.006 *	4.1	2.3	0.000 *	4.2	2.5	<0.0001 *
Price	4.0	2.9	0.000 *	3.0	2.9	0.496	3.8	3.2	0.011 *	4.2	2.6	<0.0001 *	4.0	3.1	0.000 *
Visual app	3.3	2.3	0.108	3.9	3.1	0.006 *	4.2	3.5	0.001 *	4.3	2.5	<0.0001 *	4.1	2.8	0.001 *
Wt. control	4.0	3.1	0.000 *	3.8	3.3	0.007 *	4.3	3.6	<0.0001 *	4.2	2.9	<0.0001 *	4.5	2.6	0.000 *
Affect regul.	1.0	1.8	0.553	3.4	2.5	0.054	3.4	2.9	0.110	4.0	2.1	0.000 *	3.3	2.2	0.182
Social norm	4.1	2.5	<0.0001 *	3.7	2.9	0.006 *	3.5	3.1	0.163	3.8	2.4	<0.0001 *	4.1	2.4	0.004 *
Social image	4.3	1.9	<0.0001 *	3.7	2.9	0.015 *	3.9	3.1	0.003 *	3.9	2.1	<0.0001 *	4.2	2.4	<0.0001 *
Choice	4.3	2.8	<0.0001 *	3.6	3.1	0.101	4.2	3.6	0.013 *	4.0	2.8	<0.0001 *	3.9	2.8	0.007 *

K = mean scores for RATA, L = mean scores for RATING, and M = *p*-values for two-sample t-test, ^1^ Five-point scale: 1 = not at all important, 2 = slightly important, 3 = moderately important, 4 = very important, and 5 = extremely important. * *p*-values were lower than the significance level alpha = 0.05, implying that particular mean scores for RATA and RATING significantly differed.

**Table A10:** Mean scores1 for RATA and RATING survey formats and *p*-values for the corresponding two-sample *t*-test for each motivation construct for fruits and vegetables in all five countries.

	Brazil			China			India			Spain			USA		
	K	L	M	K	L	M	K	L	M	K	L	M	K	L	M
Liking	4.3	4.3	0.586	4.0	3.5	<0.0001 *	4.3	3.8	<0.0001 *	4.2	4.0	0.011 *	4.2	3.9	0.006 *
Habits	3.8	3.9	0.409	3.5	3.3	0.319	4.1	3.8	0.003 *	3.6	3.4	0.213	3.7	3.4	0.054
Need/Hunger	4.2	3.9	0.001 *	3.9	3.3	0.001 *	4.2	3.8	0.000 *	4.0	3.8	0.030 *	3.9	3.7	0.185
Health	4.5	3.8	<0.0001 *	4.1	3.5	<0.0001 *	4.5	3.9	<0.0001 *	4.3	3.8	<0.0001 *	4.3	3.5	<0.0001 *
Convenience	3.9	3.6	0.094	3.4	3.3	0.568	3.8	3.4	0.089	4.1	3.4	0.000 *	3.6	3.7	0.931
Pleasure	4.0	3.3	0.000 *	3.2	3.1	0.579	4.1	3.7	0.013 *	4.1	3.6	0.000 *	4.0	3.4	0.000 *
Trad. eating	3.8	2.7	<0.0001 *	3.4	3.0	0.175	4.0	3.5	0.002 *	4.0	2.9	<0.0001 *	3.2	2.7	0.064
Nat. concern	4.5	3.9	<0.0001 *	4.0	3.5	0.001 *	4.4	3.9	<0.0001 *	4.3	3.7	0.000 *	4.2	3.4	<0.0001 *
Sociability	3.7	2.3	0.115	4.2	2.9	0.000 *	3.8	3.1	0.006 *	3.7	2.2	0.000 *	4.4	2.4	0.004 *
Price	4.1	3.1	<0.0001 *	3.4	2.9	0.040 *	3.9	3.1	<0.0001 *	3.9	2.7	0.000 *	4.2	3.1	0.000 *
Visual app	3.7	2.4	0.017 *	3.8	3.0	0.007 *	4.1	3.3	<0.0001 *	4.1	2.7	<0.0001 *	4.2	2.7	<0.0001 *
Wt. control	4.4	3.3	<0.0001 *	3.7	3.3	0.019 *	4.3	3.5	<0.0001 *	4.1	3.0	<0.0001 *	4.0	3.1	<0.0001 *
Affect regul.	1.0	1.8	0.554	3.7	2.6	0.036 *	3.1	2.9	0.483	4.2	2.0	0.000 *	3.5	2.2	0.041 *
Social norm	3.7	2.4	<0.0001 *	3.9	2.9	0.000 *	3.2	3.0	0.412	3.8	2.4	0.000 *	4.5	2.5	<0.0001 *
Social image	3.5	1.9	0.025 *	3.7	3.0	0.023 *	3.6	3.0	0.020 *	4.4	2.0	<0.0001 *	4.1	2.3	<0.0001 *
Choice	4.0	2.9	0.000 *	3.4	3.0	0.296	4.0	3.4	0.008 *	3.8	2.8	0.006 *	4.0	2.8	0.003 *

K = mean scores for RATA, L = mean scores for RATING, and M = *p*-values for two-sample t-test, ^1^ Five-point scale: 1 = not at all important, 2 = slightly important, 3 = moderately important, 4 = very important, and 5 = extremely important. * *p*-values were lower than the significance level alpha = 0.05, implying that particular mean scores for RATA and RATING significantly differed.

**Table A11:** Mean scores1 for RATA and RATING survey formats and *p*-values for the corresponding two-sample *t*-test for each motivation construct for starch-rich foods in all five countries.

	Brazil			China			India			Spain			USA		
	K	L	M	K	L	M	K	L	M	K	L	M	K	L	M
Liking	4.2	4.0	<0.0001 *	3.7	3.6	0.263	4.2	3.8	<0.0001 *	4.1	4.0	0.083	4.1	4.0	0.048 *
Habits	3.6	3.7	0.054	3.5	3.7	0.038 *	3.9	3.8	0.140	3.3	3.0	0.003 *	3.2	3.2	0.711
Need/Hunger	4.3	3.6	<0.0001 *	3.9	3.7	0.004 *	4.1	3.6	<0.0001 *	3.9	3.6	0.000 *	3.9	3.6	0.006 *
Health	4.3	3.2	<0.0001 *	4.1	3.4	<0.0001 *	4.3	3.6	<0.0001 *	4.0	3.0	<0.0001 *	4.0	2.9	<0.0001 *
Convenience	4.0	3.3	<0.0001 *	3.4	3.4	0.726	4.1	3.8	0.000 *	3.7	2.7	<0.0001 *	3.7	3.3	0.000 *
Pleasure	4.0	3.0	<0.0001 *	3.5	3.0	0.014 *	4.2	3.6	<0.0001 *	4.0	3.6	<0.0001 *	4.2	3.4	<0.0001 *
Trad. eating	3.5	2.7	<0.0001 *	3.5	3.4	0.231	4.0	3.6	<0.0001 *	3.6	3.1	<0.0001 *	3.0	2.8	0.067
Nat. concern	4.4	3.4	<0.0001 *	4.1	3.5	0.000 *	4.3	3.8	<0.0001 *	4.3	3.3	<0.0001 *	4.1	3.1	<0.0001 *
Sociability	3.9	2.6	<0.0001 *	3.8	3.0	0.001 *	3.9	3.3	0.000 *	3.8	2.9	<0.0001 *	3.9	2.3	0.000 *
Price	4.1	2.9	<0.0001 *	3.5	2.9	0.009 *	3.9	3.1	<0.0001 *	4.0	2.4	<0.0001 *	3.9	3.0	<0.0001 *
Visual app	3.5	2.2	<0.0001 *	3.6	3.0	0.023 *	4.0	3.3	<0.0001 *	3.6	2.6	<0.0001 *	3.3	2.7	0.019 *
Wt. control	4.2	2.9	<0.0001 *	3.7	3.1	0.002 *	4.2	3.4	<0.0001 *	4.1	2.6	<0.0001 *	3.9	2.7	<0.0001 *
Affect regul.	5.0	1.6	0.003 *	2.5	2.2	0.370	3.1	2.7	0.108	4.3	1.8	0.000 *	4.3	2.0	<0.0001 *
Social norm	3.7	2.3	<0.0001 *	3.3	2.8	0.012 *	3.8	2.9	<0.0001 *	3.6	2.3	<0.0001 *	4.1	2.3	<0.0001 *
Social image	2.8	1.8	0.003 *	3.4	2.8	0.021 *	3.7	3.0	<0.0001 *	3.7	2.1	<0.0001 *	4.4	2.2	<0.0001 *
Choice	3.9	2.8	<0.0001 *	3.6	3.1	0.006 *	3.9	3.3	0.001 *	3.9	2.3	<0.0001 *	3.8	2.6	0.000 *

K = mean scores for RATA, L = mean scores for RATING, and M = *p*-values for two-sample *t*-test, ^1^ Five-point scale: 1 = not at all important, 2 = slightly important, 3 = moderately important, 4 = very important, and 5 = extremely important. * *p*-values were lower than the significance level alpha = 0.05, implying that particular mean scores for RATA and RATING significantly differed.

**Table A12:** Mean scores1 for RATA and RATING survey formats and *p*-values for the corresponding two-sample *t*-test for each motivation construct for dessert foods in all five countries.

	Brazil			China			India			Spain			USA		
	K	L	M	K	L	M	K	L	M	K	L	M	K	L	M
Liking	4.3	4.2	0.115	3.9	3.4	0.003 *	4.2	3.7	<0.0001 *	4.1	3.9	0.069	4.3	3.8	<0.0001 *
Habits	3.8	3.4	0.064	3.2	3.4	0.540	4.1	3.7	0.004 *	3.9	3.3	0.002 *	4.4	3.2	<0.0001 *
Need/Hunger	4.2	3.1	<0.0001 *	4.0	3.4	0.004 *	4.1	3.5	0.000 *	4.0	3.2	0.005 *	3.7	3.3	0.137
Health	4.4	2.3	<0.0001 *	3.9	3.1	0.012 *	4.1	3.2	<0.0001 *	4.0	2.5	0.001 *	4.9	2.5	<0.0001 *
Convenience	4.3	3.0	<0.0001 *	3.4	3.2	0.632	3.7	3.2	0.021 *	4.3	2.9	0.001 *	4.7	2.9	<0.0001 *
Pleasure	3.9	3.5	0.042 *	4.2	3.3	0.001 *	4.2	3.7	0.000 *	4.0	3.8	0.057	3.9	3.7	0.315
Trad. eating	3.7	2.8	0.000 *	3.2	3.2	0.961	4.1	3.4	0.000 *	3.9	3.5	0.001 *	3.5	3.0	0.058
Nat. concern	4.6	2.3	<0.0001 *	3.9	3.3	0.225	4.2	3.2	<0.0001 *	4.2	3.0	0.012 *	4.6	2.7	0.000 *
Sociability	4.0	3.0	<0.0001 *	3.7	3.2	0.178	3.8	3.3	0.010 *	3.6	2.9	0.007 *	3.7	2.7	0.009 *
Price	4.4	2.6	0.000 *	3.7	3.0	0.046 *	3.9	3.0	0.000 *	3.6	2.3	0.039 *	4.2	2.7	0.001 *
Visual app	3.9	2.9	0.003 *	4.2	3.1	0.000 *	4.2	3.5	<0.0001 *	4.0	2.8	0.001 *	4.0	3.0	0.010 *
Wt. control	4.7	2.1	0.002 *	3.6	3.1	0.113	3.9	3.1	0.001 *	4.3	2.3	0.011 *	4.3	2.4	0.014 *
Affect regul.	3.8	2.4	<0.0001 *	3.5	3.0	0.317	3.6	3.1	0.041 *	4.4	2.2	0.000 *	3.8	2.5	0.053
Social norm	4.0	2.3	0.257	4.0	3.2	0.064	3.5	3.0	0.112	4.1	2.4	<0.0001 *	4.4	2.6	0.001 *
Social image	4.3	2.1	0.003 *	3.9	3.1	0.017 *	3.8	3.2	0.030 *	4.3	2.1	<0.0001 *	4.4	2.5	<0.0001 *
Choice	4.6	2.5	0.000 *	3.9	3.2	0.105	4.0	3.3	0.054	4.3	2.5	0.012 *	3.5	2.7	0.112

K = mean scores for RATA, L = mean scores for RATING, and M = p-values for two-sample t-test, ^1^ Five-point scale: 1 = not at all important, 2 = slightly important, 3 = moderately important, 4 = very important, and 5 = extremely important. * *p*-values were lower than the significance level alpha = 0.05, implying that particular mean scores for RATA and RATING significantly differed.

## References

[1] Seninde D., Chambers E.I. (2020). A Comparison of the Percentage of “Yes” (Agree) Responses and Importance of Attributes (Constructs) determined using Check-All-That-Apply and Check-All-Statements (Yes/No) Question Formats in Five Countries. Foods.

[2] Jaeger S.R., Cadena R.S., Torres-Moreno M., Antúnez L., Vidal L., Giménez A., Hunter D.C., Beresford M.K., Kam K., Yin D. (2014). Comparison of check-all-that-apply and forced-choice Yes/No question formats for sensory characterisation. Food Qual. Prefer..

[3] Vidal L., Ares G., Hedderley D.I., Meyners M., Jaeger S.R. (2018). Comparison of rate-all-that-apply (RATA) and check-all-that-apply (CATA) questions across seven consumer studies. Food Qual. Prefer..

[4] Smyth J.D., Dillman D.A., Christian L.M., Stern M.J. (2006). Comparing check-all and forced-choice question formats in Web surveys. Public Opin. Q..

[5] Smyth J.D., Christian L.M., Dillman D.A. (2008). Does “yes or no” on the telephone mean the same as “check-all-that-apply” on the web?. Public Opin. Q..

[6] Nicolaas G., Campanelli P., Hope S., Jäckle A., Lynn P. (2015). Revisiting “yes/no” versus “check all that apply”: Results from a mixed modes experiment. Surv. Res. Methods.

[7] Seninde D.R., Chambers E. (2020). Comparing Four Question Formats in Five Languages for On-Line Consumer Surveys. Methods Protoc..

[8] Likert R. (1932). A technique for the measurement of attitudes. Arch. Psychol..

[9] Raspa M., Wylie A., Wheeler A.C., Kolacz J., Edwards A., Heilman K., Porges S.W. (2018). Sensory Difficulties in Children With an FMR1 Premutation. Front. Genet..

[10] Harland N.J., Dawkin M.J., Martin D. (2014). Relative utility of a visual analogue scale vs. a six-point Likert scale in the measurement of global subject outcome in patients with low back pain receiving physiotherapy. Physiotherapy.

[11] Jaeger S.R., Lee S.M., Kim K.O., Chheang S.L., Roigard C.M., Ares G. (2018). CATA and RATA questions for product-focused emotion research: Five case studies using emoji questionnaires. Food Qual. Prefer..

[12] Meyners M., Jaeger S.R., Ares G. (2016). On the analysis of Rate-All-That-Apply (RATA) data. Food Qual. Prefer..

[13] Jaeger S.R., Ares G. (2015). RATA questions are not likely to bias hedonic scores. Food Qual. Prefer..

[14] Ares G., Bruzzone F., Vidal L., Cadena R.S., Giménez A., Pineau B., Hunter D.C., Paisley A.G., Jaeger S.R. (2014). Evaluation of a rating-based variant of check-all-that-apply questions: Rate-all-that-apply (RATA). Food Qual. Prefer..

[15] Ares G., de Andrade J.C., Antúnez L., Alcaire F., Swaney-Stueve M., Gordon S., Jaeger S.R. (2017). Hedonic product optimisation: CATA questions as alternatives to JAR scales. Food Qual. Prefer..

[16] Jaeger S.R., Fiszman S., Reis F., Chheang S.L., Kam K., Pineau B., Deliza R., Ares G. (2017). Influence of evoked contexts on hedonic product discrimination and sensory characterizations using CATA questions. Food Qual. Prefer..

[17] Jaeger S.R., Kim K.O., Lee S.M., Hunter D.C., Kam K., Chheang S.L., Jin D., Lee P.Y., Xia Y.X., Ares G. (2017). Concurrent elicitation of hedonic and CATA/RATA responses with Chinese and Korean consumers: Hedonic bias is unlikely to occur. Food Qual. Prefer..

[18] Jaeger S.R., Swaney-Stueve M., Chheang S.L., Hunter D.C., Pineau B., Ares G., Jaeger S.R., Smyth J.D., Dillman D.A., Christian L.M. (2014). An assessment of the CATA-variant of the EsSense Profile^®^. Food Qual. Prefer..

[19] Sudman S., Bradburn N.M., Bradburn N.M. (1982). Asking Questions.

[20] Yeh L.L., Kim K.O., Chompreeda P., Rimkeeree H., Yau N.J.N., Lundahl D.S. (1998). Comparison in Use of the 9-Point Hedonic Scale between Americans, Chinese, Koreans, and Thai. Food Qual. Prefer..

[21] Yao E., Lim J., Tamaki K., Ishii R., Kim K.O., O’Mahony M. (2003). Structured and unstructured 9-point hedonic scales: A cross cultural study with American, Japanese and Korean consumers. J. Sens. Stud..

[22] Cox D.N., Clark M.R., Mialon V.S. (2001). A cross-cultural methodological study of the uses of two common hedonic response scales. Food Qual. Prefer..

[23] Schwarz N. (1999). Self-Reports: How the Questions Shape the Answers. Am. Psychol..

[24] Spector P.E. (1980). Ratings of Equal and Unequal Response Choice Intervals. J. Soc. Psychol..

[25] Spector P.E. (1992). Summated Rating Scale Construction Vol. 82: An Introduction.

[26] Stevens S.S. (1968). Measurement, Statistics, and the Schemapiric View. Sci. Am. Assoc. Adv. Sci..

[27] Jones L.V., Peryam D.R., Thurstone L.L. (1955). Development of a scale for measuring soldiers’ food preferences. Food Res..

[28] Preston C.C., Colman A.M. (2000). Optimal number of response categories in rating scales: Reliability, validity, discriminating power, and respondent preferences. Acta Psychol. (Amst.).

[29] Schaeffer N.C., Dykema J. (2020). Advances in the Science of Asking Questions. Annu. Rev. Sociol..

[30] Andriosopoulos K., Bigerna S., Bollino C.A., Micheli S. (2018). The impact of age on Italian consumers’ attitude toward alternative fuel vehicles. Renew. Energy.

[31] Chang K.J., Liz Thach M.W., Olsen J. (2016). Wine and health perceptions: Exploring the impact of gender, age and ethnicity on consumer perceptions of wine and health. Wine Econ. Policy.

[32] Hartley J. (2014). Some thoughts on Likert-type scales. Int. J. Clin. Heal. Psychol..

[33] Cohen L., Manion L. (1980). Research Methods in Education.

[34] Knapp T.R. (1990). Treating ordinal scales as interval scales: An attempt to resolve the controversy. Nurs. Res..

[35] Kuzon W M J., Urbanchek M.G., McCabe S. (1996). The seven deadly sins of statistical analysis. Ann. Plast. Surg..

[36] Jamieson S. (2004). Likert scales: How to (ab)use them. Med. Educ..

[37] Doering T.R., Hubbard R. (1979). Measurement and Statistics: The Ordinal-Interval Controversy and Geography. Area.

[38] Sieber J.E., Stanley B. (1988). Ethical and Professional Dimensions of Socially Sensitive Research. Am. Psychol..

[39] Groves R.M. (1989). Survey Errors and Survey Costs.

[40] Schouteten J.J., Gellynck X., Slabbinck H. (2019). Influence of organic labels on consumer’s flavor perception and emotional profiling: Comparison between a central location test and home-use-test. Food Res. Int..

[41] Ng M., Chaya C., Hort J. (2013). Beyond liking: Comparing the measurement of emotional response using EsSense Profile and consumer defined check-all-that-apply methodologies. Food Qual. Prefer..

[42] Groves R.M. (2011). THREE ERAS OF SURVEY RESEARCH. Public Opin. Q..

[43] Hoonakker P., Carayon P. (2009). Questionnaire Survey Nonresponse: A Comparison of Postal Mail and Internet Surveys. Int. J. Hum. Comput. Interact..

[44] Lavrakas P. (2013). Internet Surveys. Encyclopedia of Survey Research Methods.

[45] Castro M., Chambers E. (2019). Willingness to eat an insect based product and impact on brand equity: A global perspective. J. Sens. Stud..

[46] Link M.W., Murphy J., Schober M.F., Buskirk T.D., Childs J.H., Tesfaye C.L. (2014). Mobile technologies for conducting, augmenting and potentially replacing surveys: Executive summary of the aapor task force on emerging technologies in public opinion research. Public Opin. Q..

[47] Conrad F.G., Schober M.F., Coiner T. (2007). Bringing features of human dialogue to web surveys. Appl. Cogn. Psychol..

[48] Phan U.T.X., Chambers E. (2016). Motivations for choosing various food groups based on individual foods. Appetite.

[49] Phan U.T.X., Chambers E. (2016). Application of An Eating Motivation Survey to Study Eating Occasions. J. Sens. Stud..

[50] Jaeger S.R., Beresford M.K., Paisley A.G., Antúnez L., Vidal L., Cadena R.S., Giménez A., Ares G. (2015). Check-all-that-apply (CATA) questions for sensory product characterization by consumers: Investigations into the number of terms used in CATA questions. Food Qual. Prefer..

[51] National Health and Medical Research Council The Five Food Groups|Eat For Health. https://www.eatforhealth.gov.au/food-essentials/five-food-groups.

[52] Curtarelli M., van Houten G. (2018). Questionnaire translation in the European company survey: Conditions conducive to the effective implementation of a TRAPD-based approach. Transl. Interpret..

[53] Harkness J.A., Harkness J.A., Van de Vijver F.J.R., Mohler P.P. (2003). Questionnaire Translation. Cross-Cultural Survey Methods.

[54] Le K.N., Tam V.W.Y. (2007). A Survey on Effective Assessment Methods to Enhance Student Learning. Australas. J. Eng. Educ..

[55] Armas C., Ordiales R., Pugnaire F.I. (2004). Measuring plant interactions: A new comparative Index. Ecology.

[56] Chambers D., Phan U., Chanadang S., Maughan C., Sanchez K., Di Donfrancesco B., Gomez D., Higa F., Li H., Chambers E. (2016). Motivations for Food Consumption during Specific Eating Occasions in Turkey. Foods.

[57] Bruzzone F., Vidal L., Antúnez L., Giménez A., Deliza R., Ares G. (2015). Comparison of intensity scales and CATA questions in new product development: Sensory characterisation and directions for product reformulation of milk desserts. Food Qual. Prefer..

[58] Peña-López I. (1999). Falling through the Net: Defining the Digital Divide..

[59] Muñoz A., King S. (2007). International Consumer Product Testing Across Cultures and Countries.

[60] Armstrong B., Reynolds C., Reynolds C., Bridge G., Oakden L., Wang C., Panzone L., Rivera X.S., Kause A., Ffoulkes C. (2021). How Does Citizen Science Compare to Online Survey Panels? A Comparison of Food Knowledge and Perceptions Between the Zooniverse, Prolific and Qualtrics UK Panels. Front. Sustain. Food Syst..

